# BUTTERFLY: addressing the pooled amplification paradox with unique molecular identifiers in single-cell RNA-seq

**DOI:** 10.1186/s13059-021-02386-z

**Published:** 2021-06-08

**Authors:** Johan Gustafsson, Jonathan Robinson, Jens Nielsen, Lior Pachter

**Affiliations:** 1 Department of Biology and Biological Engineering, Chalmers University of Technology, Kemivägen 10, Gothenburg, Sweden; 2 Wallenberg Center for Protein Research, Chalmers University of Technology, Kemivägen 10, Gothenburg, Sweden; 3 Department of Biology and Biological Engineering, National Bioinformatics Infrastructure Sweden, Science for Life Laboratory, Chalmers University of Technology, Kemivägen 10, SE-41258 Gothenburg, Sweden; 4 BioInnovation Institute, Ole Maaløes Vej 3, DK2200 Copenhagen N, Denmark; 5 Division of Biology and Biological Engineering & Department of Computing and Mathematical Sciences, California Institute of Technology, Pasadena, USA

**Keywords:** Single-cell RNA-Seq, UMI, Droplet-based, PCR, Bias, Amplification, Batch correction, Correction

## Abstract

The incorporation of unique molecular identifiers (UMIs) in single-cell RNA-seq assays makes possible the identification of duplicated molecules, thereby facilitating the counting of distinct molecules from sequenced reads. However, we show that the naïve removal of duplicates can lead to a bias due to a “pooled amplification paradox,” and we propose an improved quantification method based on unseen species modeling. Our correction called BUTTERFLY uses a zero truncated negative binomial estimator implemented in the kallisto bustools workflow. We demonstrate its efficacy across cell types and genes and show that in some cases it can invert the relative abundance of genes.

**Supplementary Information:**

The online version contains supplementary material available at 10.1186/s13059-021-02386-z.

## Background

Droplet-based single-cell RNA sequencing (scRNA-Seq) technologies have made possible quantification of transcriptomes in individual cells at a large scale [1], enabling the study of the diversity in molecular state among cells. As an increasing number of datasets have been collected [2], methods for integration of results derived by different laboratories have become paramount. The diversity of experimental and computational methods used in producing individual datasets makes careful accounting of technical and batch effects essential [3].

**Fig. 1:**
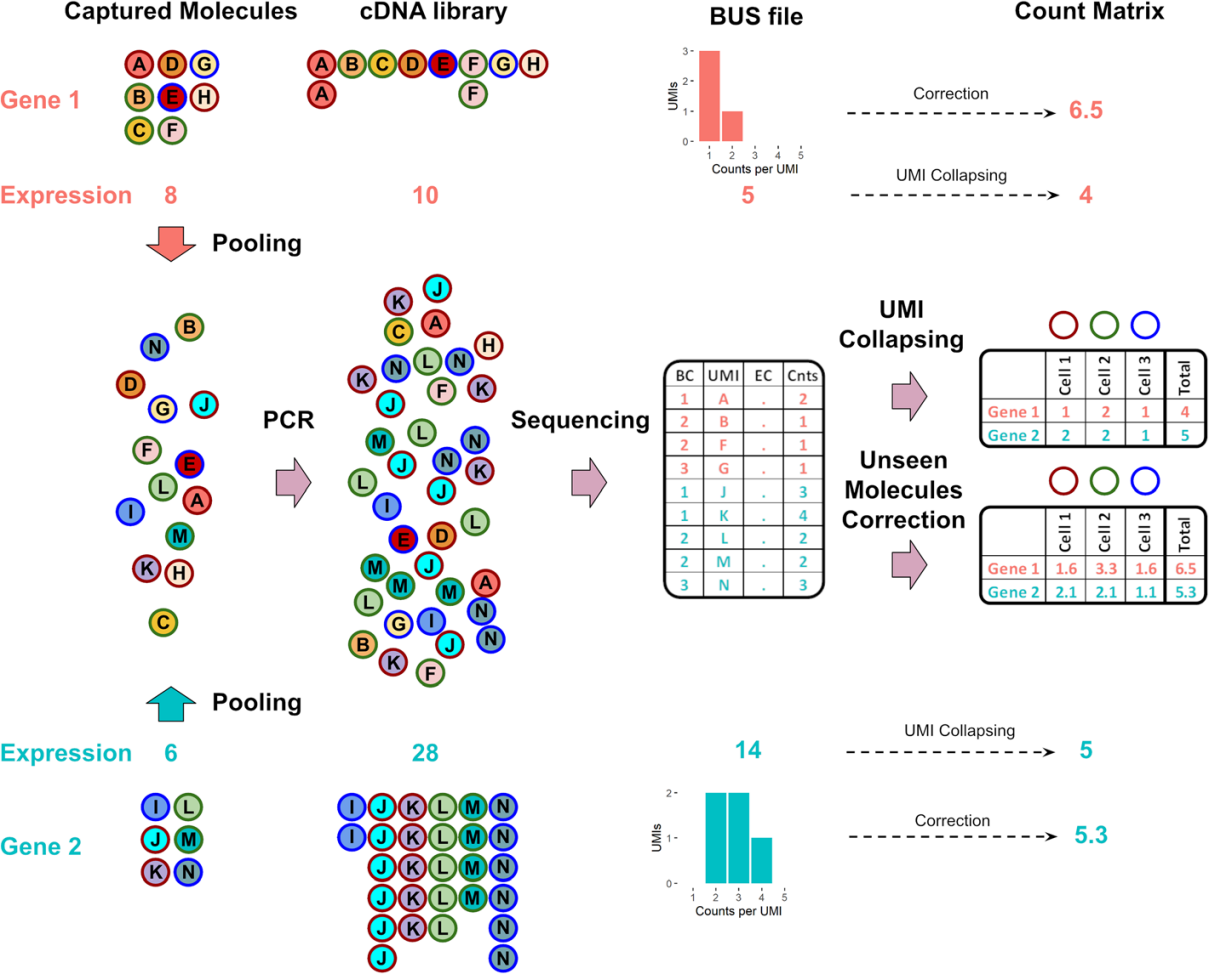
The pooled amplification paradox. Illustration of a seemingly paradoxical reversal in gene abundance estimates arising from incomplete sampling of a cDNA library generated after a differential amplification of two genes. Consider a situation where more molecules have been captured for Gene 1 (8) than Gene 2 (6). Despite the correction for amplification bias with unique molecular identifiers, the increased amplification of Gene 2 relative to Gene 1, coupled with the incomplete sequencing of the cDNA library, results in a lower copy number estimate of Gene 1 (4) in comparison to Gene 2 (5). Correction of abundance estimates due to unseen molecules using a histogram of counts per UMI improves the quantification, reversing the relative amount of Gene 1 and Gene 2

Most single-cell RNA-seq technologies require amplification of the RNA starting material via PCR, a step that is known to introduce bias across genes depending on the nucleotide sequence. For example, amplification has been shown to depend on the GC content of a gene [4]. Single-cell RNA-Seq can require many PCR amplification cycles, even more than bulk sequencing, due to the small amount of mRNA molecules available in each cell. Fortunately, the introduction of unique molecular identifiers (UMIs) [5], where all mRNA molecules are tagged with random barcodes, can be used to account for PCR duplicates, since copies of captured mRNA molecules can be detected and discarded. This process is known as deduplication or UMI collapsing [6]. However, UMI collapsing does not address another bias that can result from incomplete representation of differentially amplified molecules in sequenced products from a library (Fig. 1), and that is common in droplet-based single-cell RNA-Seq experiments [7]. In extreme cases, the differential amplification of pooled molecules that are sequenced together can result in inversion of relative abundances, leading to a pooled amplification paradox.

Estimation of unseen species [8, 9] is commonly used in ecology, where the number of species encountered in an ecosystem given a certain sampling effort is estimated from a limited number of samples. We translate this to the problem of estimating the unseen number of molecules in a single-cell sequencing experiment, where the species correspond to unique molecules and samples to sequencing reads. We show that this is possible in assays that utilize unique molecular identifiers.

The mathematical problem of estimating unseen species has previously been addressed with the introduction of several estimators. The simplest is the Good-Toulmin estimator [8], which while easy to utilize, is limited in prediction range, producing stable predictions of species represented only in up to twice the number of samples. Although this limitation can be addressed [10], the estimator is still not practically useful for sequencing experiments. Daley et al. therefore developed Preseq [11–13], which does not suffer from such limitations, and employed it to estimate the optimal number of reads for genome sequencing. Their method is based on rational function approximation, which substantially increases the radius of convergence of the power series appearing in the Good-Toulmin estimator, providing a stable estimator.

Here, we present BUTTERFLY, a method that utilizes estimation of unseen species for addressing the bias caused by incomplete sampling of differentially amplified molecules. Specifically, we extrapolate the gene expression to a higher number of reads by estimating the missing number of molecules per gene, validate the results using two different approaches, and demonstrate the utility of the method for batch correction. We also show that it can mitigate identification of false positive cell type markers. BUTTERFLY is implemented in C++ as part of the kallisto bustools single-cell RNA-seq workflow [14].

## Results

**Fig. 2:**
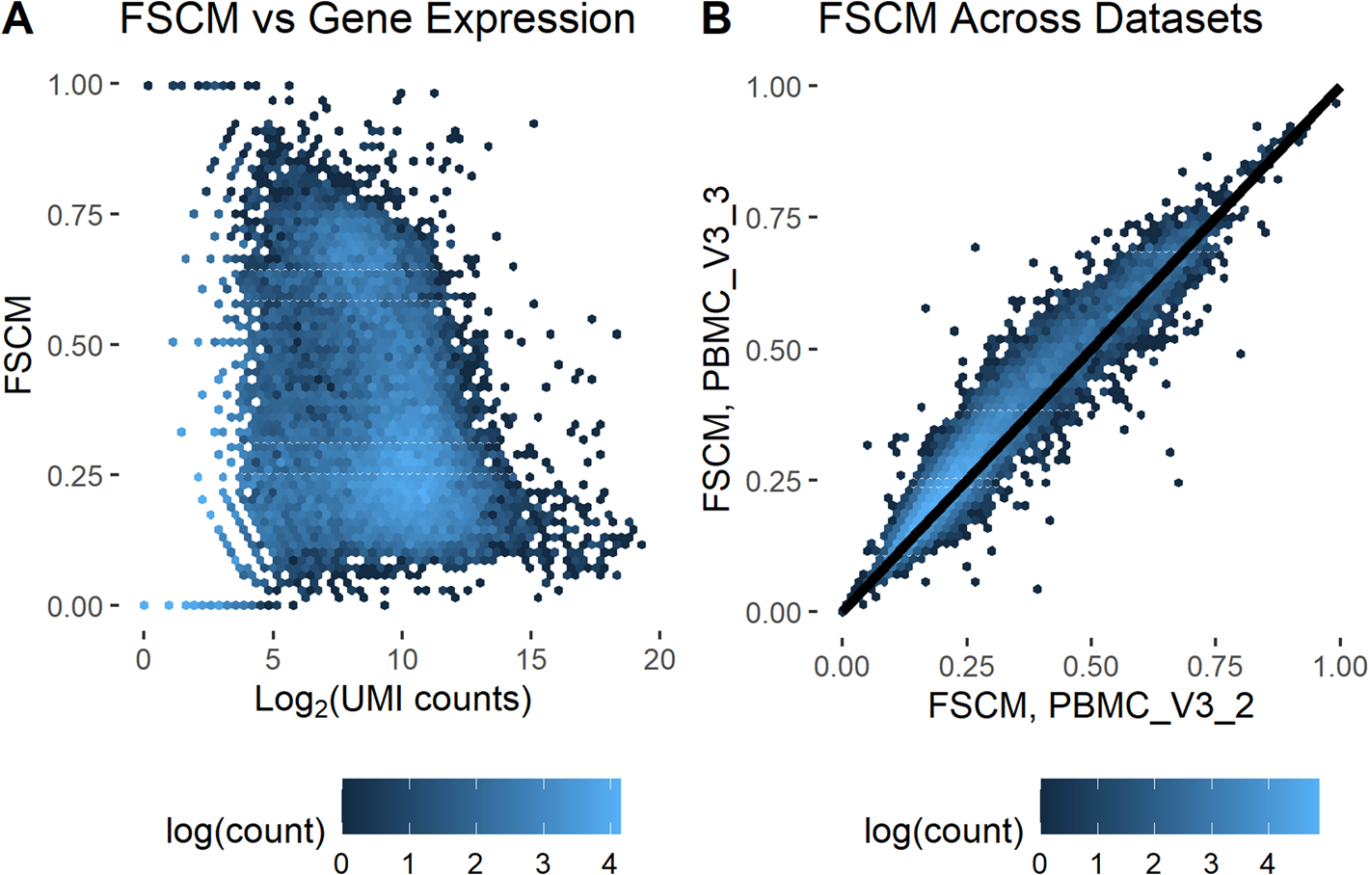
Differences in fraction of single-copy molecules across genes. **A** Fraction of single copy molecules (FSCM) vs mean gene expression across cells for the PBMC_V3_3 dataset. **B** Comparison of gene FSCM between 2 datasets (PBMC_V3_2 and PBMC_V3_3) produced using the same technology (10x Chromium v3). The code to reproduce this figure is here: https://github.com/pachterlab/GRNP_2020/blob/master/notebooks/figure_generation/GenFig2_S4_S5.ipynb

To measure the extent and heterogeneity of amplification bias, we analyzed a total of 14 scRNA-seq datasets, which were generated from a total of 6 different technologies (see the “Methods” section). We started by measuring the fraction of single-copy molecules (FSCM) per gene in datasets, since it provides a useful way to summarize the extent of amplification with a single number. We found that the variation in RNA amplification was substantial across genes (Additional file 1: Fig S1–S3). Amplification bias patterns were different across technologies. For example, all 10X technologies (Additional file 1: Fig. S1) displayed consistently more amplification bias than Drop-seq (Additional file 1: Fig. S2), Seq-Well, or MARS-Seq 2.0 (Additional file 1: Fig. S3). Furthermore, we found that amplification bias is consistent across technologies. For example, the variance in FSCM in 10X technologies was consistently 0.033 (std. dev. 0.012) whereas the variance in FSCM in the Drop-seq datasets was 0.018 (std. dev. 0.002), a stark difference even considering that the datasets have varying sequencing depth. We also found that specific genes were more likely to be affected by amplification bias than others (Fig. 2A, Additional file 1: Fig. S4). Interestingly, the genes with high amplification bias, as determined by their FSCM, are generally the same across datasets, especially in data generated with the same technology (Fig. 2B, Additional file 1: Fig. S5). There is a weak association between amplification and both GC content and gene length (Additional file 1: Fig. S6), where genes with very low GC content seem to amplify less well and shorter transcripts are more amplified than their longer counterparts. FSCM values for genes are provided in Additional file 2: Table S1.

**Fig. 3:**
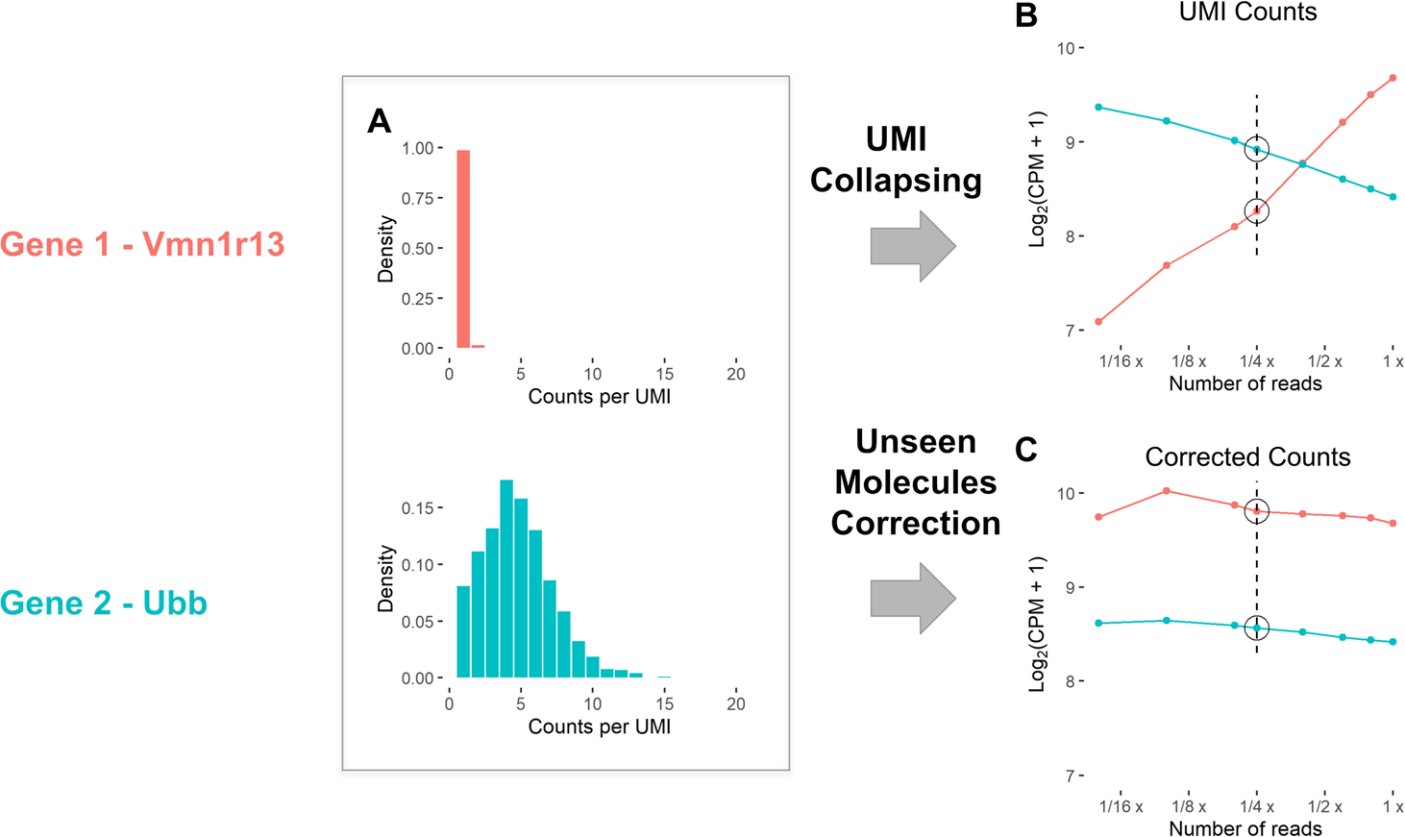
Example of the pooled amplification paradox as seen in an analysis of the EVAL dataset. **A** Histograms of the counts per UMI for two genes with different amplification. The histograms are generated from downsampled data (4 times), visualized by a dashed line in **B** and **C**. **B** Change in the mean gene expression estimates across cells of the two genes when downsampling the reads. **C** Change in gene expression for downsampled data when correcting for unseen molecules. The code to reproduce this figure is here: https://github.com/pachterlab/GRNP_2020/blob/master/notebooks/figure_generation/GenFig1_3.ipynb

To assess the implications of amplification bias on single-cell RNA-seq quantification, we downsampled reads in a mouse 10X brain cortex dataset (the EVAL dataset, see the “Methods” section) and analyzed quantification of abundances with standard UMI collapsing for a pair of genes (Fig. 3). We found that while the vomeronal 1 receptor 13 gene (*Vmn1r13*) appeared to be 2.4 times more highly expressed than the Ubiquitin B gene (*Ubb*) in the full dataset (30 M reads), when downsampling to 1.5 M reads (1/20th the dataset) the opposite was the case: *UBB* was 4.9 times more highly expressed than *vmn1r13*. Increasing the number of reads yields the discovery of many new molecules for *vmn1r13*, but few for *Ubb*, since many more of the molecules belonging to *Ubb* have already been sampled, and are therefore “canceled out” during UMI collapsing.

We hypothesized that by predicting unseen species [8, 9], we could reduce, or eliminate, the bias introduced by naive UMI collapsing. Each gene’s expected increase in gene expression with more reads can be estimated from measurements of counts per UMI (CU histogram, see Fig. 3A). We evaluated several methods for this purpose (Additional file 1: Fig. S7–S21): The Daley et al. [11] Preseq method (Preseq DS) exhibits similar performance to the zero-truncated negative binomial (ZTNB) [9], and both are more stable than the Good-Toulmin estimator [8]. We found that ZTNB has better performance for highly expressed genes, while Preseq DS performs slightly better for medium range genes for some datasets (see Additional file 1: Supplementary Note 1).

To investigate the bias theoretically, we simulated data where each molecule within a gene is amplified according to a negative binomial distribution (Additional file 1: Fig. S22 and Supplementary Note 2). First, we estimated the theoretical gene expression bias introduced at different amplification levels, where the amplification range matches that of the measured amplification in the dataset PBMC_V3_3 (Additional file 1: Fig. S22 A). For uncorrected data, the fold change between the most extreme genes is substantial, while correction (ZTNB) to a large extent corrects the bias. To determine how the bias affects the ability to detect a difference in gene expression between genes, we simulated datasets with pairs of genes where 50% of the gene pairs were expressed at different levels, and all genes have an amplification level randomly selected from the genes of the PBMC_V3_3 dataset. The area under the curve (AUC) was then calculated for the ability to from the observed gene expression identify gene pairs with different original expression levels (Additional file 1: Fig. S22 B). ZTNB correction improves this ability substantially, also at large fold change levels.

The pooled amplification bias leads to a batch effect between batches with similar amplification patterns across genes but with different read depth, since the gain of new molecules varies across genes as more reads are added. To investigate the magnitude of this bias, we simulated pairs of genes, where the amplifications of the first genes in the pairs were randomly selected from the PBMC_V3_3 dataset. The amplification (negative binomial mean) of the second gene in the pairs was set to that of the first gene, but scaled with a factor equal for all genes. We then looked at the ability to identify genes that were differentially expressed across the batches (Additional file 1: Fig. S22 C). The binomial downsampling correction (Methods), which seeks to make the batches comparable rather than aiming for the true gene expression, is very effective at removing this bias.

The gene expression of genes is important for prediction—the more molecules available, the less noisy the histogram used for prediction will be. We investigated this by simulating genes with different gene expression and again measured the ability to identify genes with different gene expression (Additional file 1: Fig. S22 D). As expected, fewer molecules lead to a decreased AUC after correction, mainly due to noisier CU histograms to use for prediction.

**Fig. 4:**
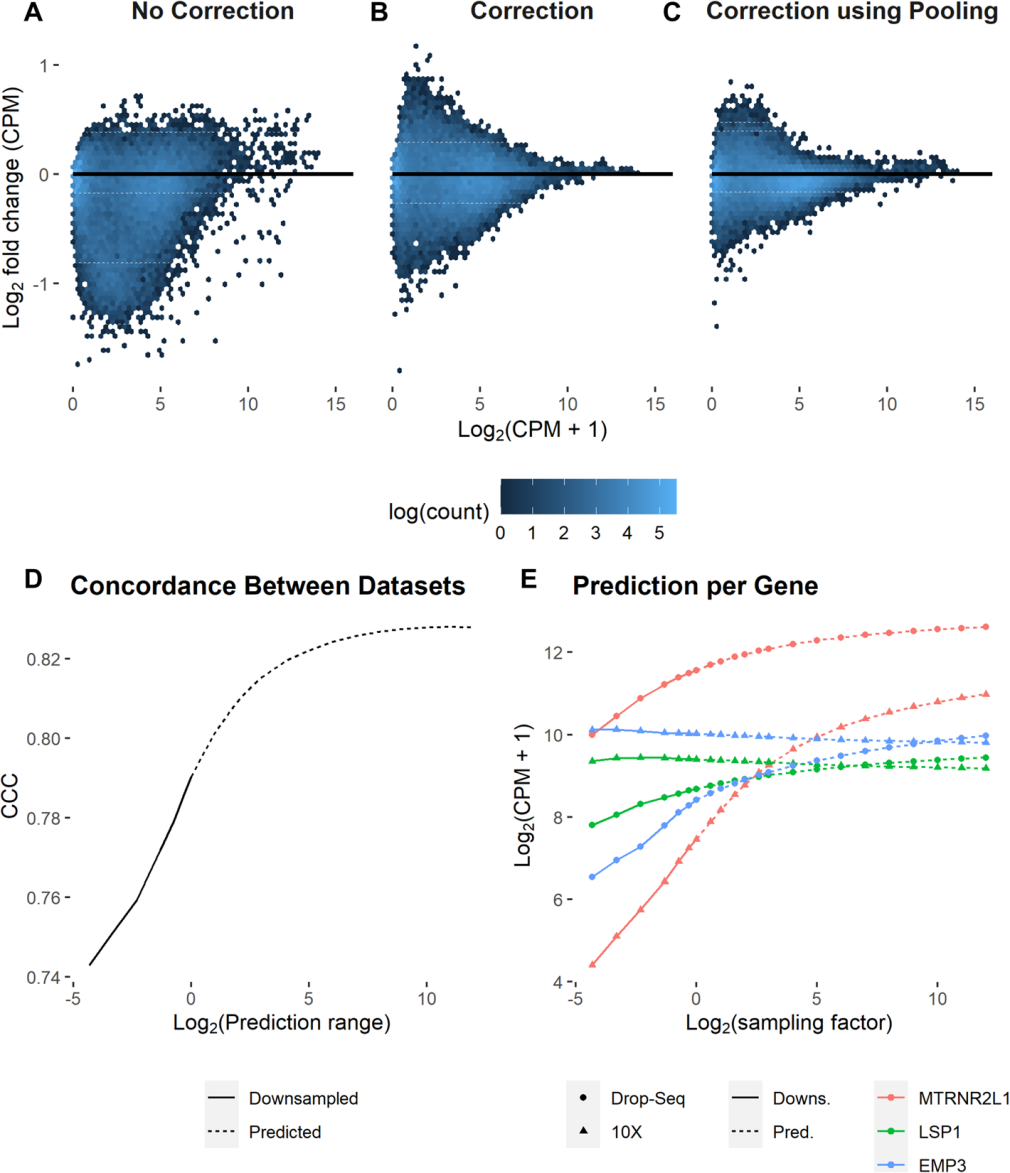
Effect of BUTTERFLY correction on gene expression. **A**–**C** The log fold change in gene expression for the PBMC_V3_3 dataset between the downsampled data (at 1/10 number of reads) and the original data. **A** No correction. **B** Unseen molecules correction applied to every gene based on the data within the dataset. **C** The same correction as in **B**, but using additional data from six other 10x Chromium datasets for estimating the copies per UMI histograms per gene (Methods). **D**, **E** Two PBMC datasets generated with different technologies (EVALPBMC, 10x Chromium v2, and EVALPBMC_DS, Drop-Seq) were downsampled to varying depths and corrected for unseen molecules using BUTTERFLY. **D** The figure shows the change in correlation (CCC) between the two datasets at different prediction/downsampling points. Both datasets are downsampled/predicted to the same degree. **E** Change in gene expression from prediction/downsampling for three genes, for both datasets. The code to reproduce this figure is here: https://github.com/pachterlab/GRNP_2020/blob/master/notebooks/figure_generation/GenFig4AC_S23.ipynb (code for **A**–**C**) and https://github.com/pachterlab/GRNP_2020/blob/master/notebooks/figure_generation/GenFig4DE.ipynb (code for **D**, **E**)

To evaluate the performance of BUTTERFLY on real data, we downsampled a human 10X peripheral blood mononuclear cell dataset (PBMC_V3_3, see the “Methods” section) to one tenth of the reads and compared the uncorrected gene expression estimates and BUTTERFLY corrected gene expression estimates in the downsampled dataset, to those of the full dataset. Figure 4A shows the log fold change for all genes where no correction is applied, showing large discrepancies for many genes. The concordance correlation coefficient (CCC) was 0.981 (see the “Methods” section), while MSE was 0.193. With BUTTERFLY the difference is clearly reduced (Fig. 4B, CCC = 0.994, MSE = 0.062), especially for highly expressed genes. These results are recapitulated in 13 other datasets from a variety of technologies including 10X Genomics v2, v3 and Next Gem, Drop-seq, SeqWell, and MARS-Seq 2.0 (Additional file 1: Fig. S8–S21). We found that pooling histograms derived from distinct datasets further improves results; we assembled CU histogram data from 6 other 10X Chromium datasets from human PBMC and utilized it for prediction (Methods). The use of pooled data clearly improves the prediction for low expression genes (Fig. 4C, CCC = 0.997, MSE = 0.030).

Part of the discrepancy between corrected expression of downsampled data and the full datasets can be explained by sampling effects. To estimate the magnitude of that effect, we compared the downsampled data with data downsampled using binomial downsampling, for which there is no sampling noise, thereby obtaining a bound on the accuracy possible (Additional file 1: Fig. S23, CCC = 0.998, MSE = 0.011).

While the downsampling results show that BUTTERFLY correction can be used to scale the gene expression of each gene to resemble the gene expression that more reads would yield, they do not necessarily imply that the corrected expression values are closer to ground truth. To investigate whether that is indeed the case, we assessed whether BUTTERFLY increases similarity of gene abundance estimates between datasets from the same biological sample assayed with different technologies. Figure 4D shows that as a correction for unseen molecules is applied to both 10X Chromium and Drop-seq datasets from PBMC cells, there is an increase in concordance. The effect of correction is highly gene dependent; for some genes the correction is modest, while it is substantial for others (Fig. 4E).

**Fig. 5:**
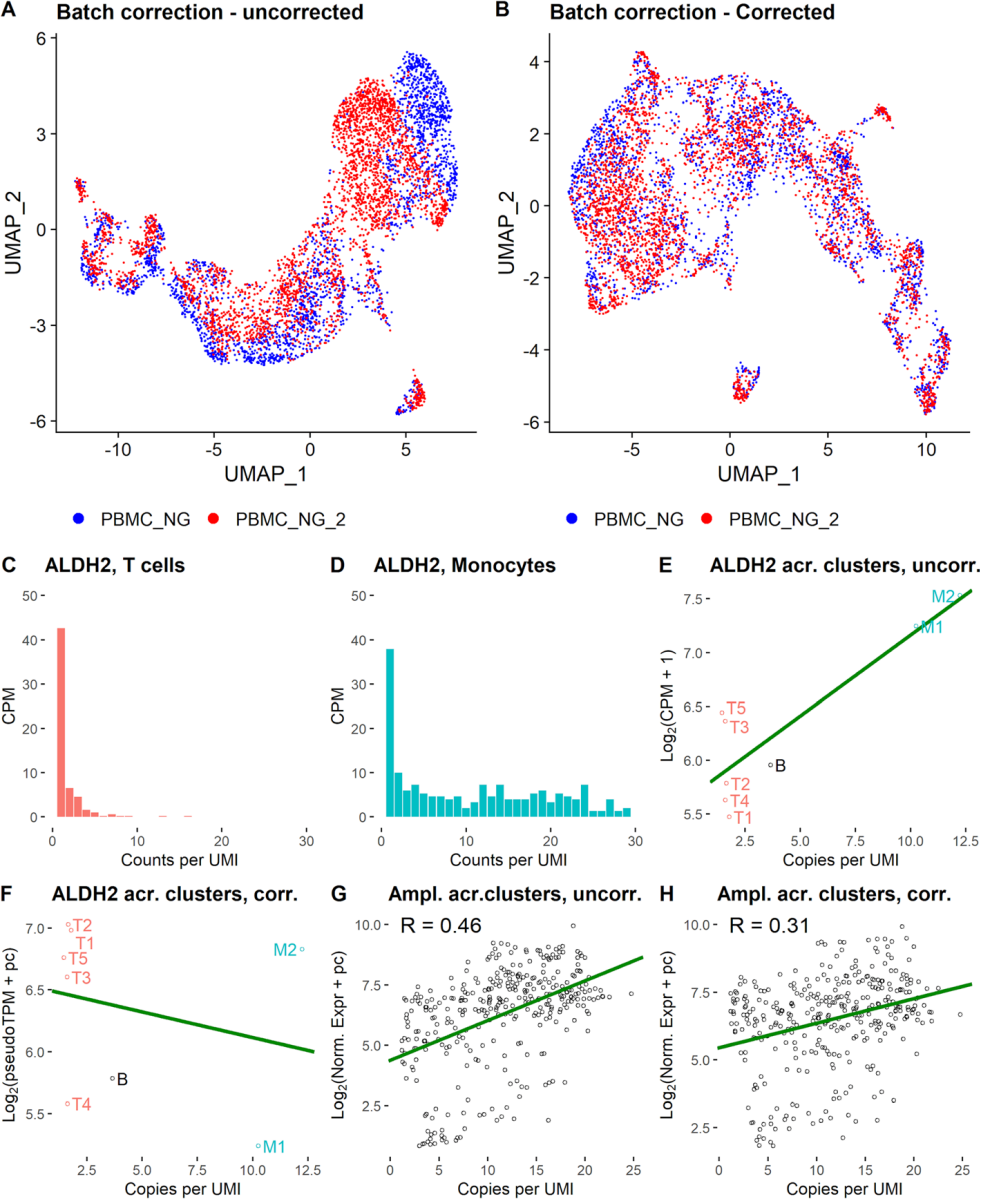
Applications of amplification bias correction. **A**, **B**. Batch correction of datasets with different sampling depths. The figures show a mix of the T cell-like cells from the PBMC_NG and PBMC_NG_2 datasets, as determined by clustering, in UMAP space. **A** Uncorrected data. **B** Data corrected using binomial downsampling of PBMC_NG_2. **C**–**H** Differences in amplification across clusters. **C** Joint CU histogram of the T cell clusters T1-T4 for the ALDH2 gene. **D** Joint CU histogram of the monocyte clusters M1-M2 for the ALDH2 gene. **E** Gene expression for the ALDH2 gene plotted against copies per UMI across clusters. **F** Corrected gene expression (using Preseq DS, predicted to 10^20^) for the ALDH2 gene plotted against copies per UMI across clusters. **G** The gene expression of the 50 genes with most variation across clusters plotted against copies per UMI, where each point represents a unique cluster and gene. **H** Similar to **G**, but for corrected data (using Preseq DS, predicted to 10^20^). No notebooks are available for the code to produce these figures, but the code is available as stated in the “Availability of data and materials” section

We also investigated the implication of BUTTERFLY for batch correction between single-cell datasets. Specifically, we investigated similar datasets (PBMC_NG and PBMC_NG_2) with different sampling depths (Fig. 5A, B). For such batch correction, prediction is suboptimal—a better alternative to prediction of the less saturated dataset for determining the gene correction is to use binomial downsampling (Methods) on the more saturated dataset. While the uncorrected data shows a clear separation by dataset (Fig. 5A), this effect is less apparent for the batch-corrected data (Fig. 5B). The correction increases the correlation between the datasets (CCC increases from 0.991 to 0.994, MSE decreases from 0.101 to 0.066), and the average fraction of the 10 nearest neighbors to each cell in UMAP space that comes from the same dataset as the cell decreases from 74 to 56%, indicating a substantially lower cell separation on dataset.

We further investigated if genes are differently amplified in different cell types. We therefore clustered the cells of the EVALPBMC dataset (Additional file 1: Fig. S24) and compared the amplification per gene across clusters, which showed large differences across clusters for some genes. Specifically, we investigated the relationship between copies per UMI and gene expression across clusters for the 50 genes with most variation in copies per UMI across cell types. For example, the gene ALDH2, which is known to be related to alcohol breakdown in the human body, shows large differences in amplification between T cells and monocytes (Fig. 5C, D). The gene appears to be more highly expressed in the clusters where it is more amplified (Fig. 5E, F-Test, p = 0.0040), although when corrected using PreSeq DS correction, the gene seems to be expressed at similar levels in the clusters (Fig. 5F, F-Test, p = 0.54). Without correction, ALDH2 could thus be identified as a marker gene for monocytes, which is mainly the result of a technical artifact in the sequencing. Across all 50 genes, there is a clear correlation between amplification and gene expression (Fig. 5G). Correcting the gene expression using the Preseq DS algorithm reduces the correlation substantially (Fig. 5H). Correction using ZTNB, for which the assumption that the data follows a negative binomial distribution is violated (for example ALDH2 for monocytes, see Fig. 5D), almost entirely removes that correlation (Additional file 1: Fig. S25). It thus seems that for some genes, a differential expression analysis that does not take amplification effects into account may find false marker genes for cell types.

## Discussion

The “pooled amplification” paradox we have highlighted is of significant concern because it can affect some genes significantly, and therefore, inferences based on their relative abundance estimates may be based on biased information. For example, the gene NEUROD1 has a very high fraction of single-copy molecules in 10x Chromium datasets (FSCM = 0.90, calculated from the joint data of all 10x PBMC datasets used in this study) suggesting that the number of cells with a detected expression of this gene is much lower than what would otherwise be expected. This gene has for example been identified as a marker gene used to identify enteroendocrine cell precursors [15]. Our results show that this gene may appear to be underrepresented in data due to low PCR amplification.

In addition to improving abundance estimates of specific genes, we have shown that BUTTERFLY can help reduce batch effects between datasets sequenced at different depths. This result should be interesting to explore in conjunction with single-cell integration methods [16]. While there exist many batch correction methods for single-cell RNA-Seq [17–19], such batch correction methods assume that differences within a cluster across datasets have a purely technical origin. Such methods risk removing biological differences across samples, while we can be certain that the BUTTERFLY binomial downsampling method only removes technical bias. Furthermore, BUTTERFLY may provide a better starting point for other batch correction methods, leaving such methods with a less challenging problem to solve.

We have shown that for some genes the amplification differs across cell types, which may lead to false discovery of cell type markers, and that BUTTERFLY helps in reducing these errors. The source of the bias across cell types is unknown, although we suspect that the differences in amplification are caused by differences in the transcript sequences. Droplet-based data do not allow for investigation of the sequences in detail, since reads are concentrated near either the 3′ or 5′ of the transcript. However, if this theory is found to be true, the amplification measurements can potentially also be used to detect differences in splice variants across cell types.

While we have demonstrated BUTTERFLY in the context of single-cell RNA-seq, the approach we have outlined is relevant for any assay in which objects are sampled after amplification, and where the pooled amplification paradox may occur (see, e.g., [20]). In particular, genomics assays utilizing UMIs should benefit from our method. There are also other applications of estimation of unseen species in genomics, as recently shown in [21].

The Preseq DS and ZTNB methods have very similar performance in our evaluation, but they still differ regarding the assumptions they make about the data. The ZTNB method assumes that the CU histogram follows a negative binomial distribution, and such an assumption is sometimes valid, for example for the Ubb gene in Fig. 3. In such a case, it will lead to a better estimate of the number of unseen molecules. However, we also see genes where this assumption is violated, for example the ALDH2 gene in monocytes in Fig. 5. Thus, while a single method is satisfactory on average, a nuanced approach with a distributional assumption mapping the data may improve results for some genes.

Our work also highlights the need for reliable estimators for the unseen species problem in the case where count histograms are based on few observations. While there has been significant effort expended in the development of estimators in the case of abundant data, this setting may be ripe for new discoveries. Furthermore, the improved reliability of estimators with more data means our approach may induce bias in accuracy favoring highly abundant genes. This should be taken into account in downstream analyses and in interpretation of results, much in the same way that improved abundance estimates of long genes affect interpretation of differential analysis [22].

## Conclusions

We have shown that naïve UMI collapsing only partially mitigates the amplification bias across genes in single-cell RNA-Seq data and that this shortcoming can lead to batch effects across datasets, incorrect quantification of gene abundances in general, and false identification of marker genes. We conducted analyses on simulated data, which highlighted the potentially substantial effects of amplification bias, and demonstrated the effect on the power to detect differences in gene expression across genes and cell types. Our BUTTERFLY correction provides an effective approach to ameliorating these biases, and our results on biological datasets show that it is beneficial to utilize it in practice. Importantly, our results highlight the importance of UMIs, not only for their utility in identifying duplicate molecules, but for addressing biases resulting from pooled amplification of differentially amplified molecules.

## Methods

### Datasets

We analyzed a total of 14 public scRNA-Seq datasets collected using 6 technologies; Drop-Seq, Seq-Well, MARS-seq 2.0, and 10x Chromium version v2, v3, and Next GEM. The datasets were generated from 5 sample types: mouse brain (dataset EVAL), mouse retina (datasets MRET and MRET2), a mix of mouse ES cells and embroynic fibroblasts (MARSSEQ), human lung tumor (dataset LC), and human PBMC (remaining datasets). The datasets, including metadata, are listed in Additional file 1: Table S2, with additional information in Additional file 1: Table S3. The datasets were processed using kallisto [23] and bustools [14], yielding counts and gene association for each unique molecule. Molecules mapped to multiple genes were discarded before analysis.

### Pre-processing of sequencing files

The datasets were processed using kallisto [23] version 0.46.2 and a version of bustools [14] specifically developed for this study (the butterfly branch), yielding counts and gene association for each unique molecule. kallisto index files were created for mouse and human using cDNA files from Ensembl (v. 96 for mouse, v. 94 for human).

We developed the new commands *collapse* and *umicorrect* in a branch of the bustools code, and modified the commands text and fromtext, to enable production of bus files mapped per gene (BUG-files) for further processing in R. The umicorrect command was implemented as described previously [24], while collapse transforms the BUS records from being mapped to equivalence classes to being merged where appropriate and mapped to genes. Transcripts to genes files, which are required for the collapse command, were generated with the function transcript2gene from the BUSpaRse R package [25], version 0.99.25.

### Processing of data in R

All reads mapping to more than one gene were discarded before further processing, as were cells with fewer than 200 UMIs (except for the LC dataset, where cells with fewer than 1000 UMIs were discarded, motivated by the large number of cells compared to the expected number from the authors). Statistical metrics were also calculated for each dataset (Additional file 1: Table S3).

### Overview of the BUTTERFLY correction

The BUTTERFLY correction is a method that supports correction of gene abundance estimates in UMI-based single-cell RNA-Seq data using either prediction of unseen species or an algorithm we have named binomial downsampling (see below).

Prediction of unseen species is used for predicting the gene expression given more reads, while binomial downsampling can be used to more accurately remove the amplification bias between two datasets with different average numbers of reads per UMI, given that they have similar amplification patterns across genes. We have evaluated the correction method using three algorithms for prediction (see below), although only the zero-truncated negative binomial (ZTNB) algorithm is implemented in the kallisto bustools workflow. The other algorithms are accessible through the R code provided with this publication.

### Correction for unseen molecules

To correct gene abundance estimates, the number of unseen molecules are predicted for each gene, assuming that the same gene behaves similarly across cells within a cell population, which if not explicitly differently specified in the text corresponds to all cells within a dataset. For each gene, all molecules across all cells within the population are pooled and used to calculate a copies per UMI (CU) histogram. The CU histogram is then in turn used as input to a prediction algorithm. Prediction was done using the Good-Toulmin estimator as well as the Daley-Smith (DS, based on rational functions approximation) and zero truncated negative binomial (ZTNB) included in the PreseqR package. We implemented the Good-Toulmin estimator in R and used the functions ds.rSAC and a modified version of ztnb.rSAC from PreseqR (where the modified version has larger error tolerances, which speeds up computation time considerably while producing very similar results). The gene expression was predicted per gene, pooling the UMIs from all cells in the dataset. Histograms over the number of copies per UMI (CU histograms) were constructed per gene and used as input to the prediction algorithms. The predicted number of UMIs per gene was then used to calculate the gene expression in counts per million (CPM). We used ZTNB for prediction except for Fig. 4C, Fig. 5F, and H, where the prediction is based on the Daley-Smith (DS) algorithm (MT = 2, described below).

#### The Good-Toulmin estimator

The Good-Toulmin estimator seeks to estimate the number of new molecules (*U*) discovered by multiplying the number of total reads with a factor *t* + 1:

$$ U=-{\sum}_{i=1}^{\infty }{\left(-t\right)}^ih(i) $$

*h(i)* here corresponds to the number of molecules with *i* copies, i.e., the value at *i* in the CU histogram. As can be seen in Additional file 1: Fig. S8–S21, the estimator performs very well for *t* ≤ 1, but is highly unstable for predictions outside that range. The Good-Toulmin estimator does not depend on any assumptions about the distribution of the data.

#### The ZTNB estimator

The ZTNB method is based on using an expectation-maximization (EM) algorithm for fitting a negative binomial curve to the histogram of number of copies per UMI, where the number of molecules with zero counts is unknown. It is then assumed that the size parameter of the negative binomial remains the same as the number of reads increase and that the mean is proportional to the number of reads. The number of molecules with at least one copy can then be estimated from the probability density function of the negative binomial.

Once the predicted gene expression is estimated, the UMI counts in the counts matrix are scaled by a factor *m*_*g*_, calculated for each gene *g* as

$$ {m}_g=\frac{T}{P}{c}_g, $$

where *T* is the total number of UMIs in the count matrix, *P* is the total number of predicted UMIs across all genes, and *c*_*g*_ is the number of predicted UMIs for gene *g*.

The EM algorithm used in Preseq for fitting the negative binomial curve to the histogram is an iterative method with two steps in each iteration: the E step and the M step. The goal is to estimate the parameters mean (μ) and size(*s*), while also taking the unseen molecules into account. In the E step, the number of molecules with zero counts, *z*, can be estimated by using the probability density function (*pdf(n,μ,s)*, *where n is the copies per molecule*) of the negative binomial at zero counts, using the current value of μ and *s*. First, the total number of molecules L is estimated:

$$ L=\frac{N}{1- pdf\left(0,\mu, s\right)} $$

where N is the total number of observed molecules. Then, the number of molecules with zero counts is calculated:

$$ z=L\  pdf\left(0,\mu, s\right) $$

The CU histogram is now complemented with the zero value, which was previously missing.

A new value for μ can then be estimated, according to

$$ \mu ={\sum}_{i=1}^{\infty }i\ h(i)/L $$

where h(i) is the number of molecules in the CU histogram at i copies. In the M step, a new value of the size parameter is then estimated using an L-BFGS-B algorithm to maximize the log likelihood. The algorithm requires a definition of the log likelihood function (*ll(s)*) and the first derivative of a function that has its maximum at the same point as the log likelihood (*pd(s)*):

$$ ll(s)={\sum}_{i=0}^{\infty }h(i)\ \mathit{\ln}\left( pdf\left(i,\mu, s\right)\right) $$

$$ pd(s)=\frac{\sum_{i=0}^{\infty } digamma\left(i+s\right)\ h(i)}{L}- digamma(s)+\mathit{\log}(s)-\mathit{\log}\left(s+\mu \right) $$

The starting values for the iteration are in the Preseq implementation selected as μ = 0.5 and *s* = 1.

The ZTNB method assumes that the data follows a negative binomial distribution. As indirectly shown in Fig. 5D, this assumption is violated for some genes since the gene is differently amplified in different cell populations, yielding a sum of several count distributions and thereby a less accurate prediction for such genes.

#### The Preseq Daley-Smith estimator

The Preseq DS estimator seeks to estimate the number of additional molecules (t) found when increasing the number of reads by a factor t + 1. The quantity is estimated as:

$$ \varDelta (t)={\sum}_{j=1}^{\infty }{\left(-1\right)}^{j+1}{\left(t-1\right)}^i{n}_j $$

(t) is however not guaranteed to converge for j > 2. To stabilize the function, an approximation is developed using rational function approximation (RFA), in which a ratio of two polynomials are used to describe the function. The Preseq DS estimator makes no assumptions about the distribution of copies per UMI within a gene. For more details, see [11].

The Preseq DS estimator has a parameter MT that can be used to truncate the CU histograms before prediction, which can stabilize the calculation. Unless otherwise stated, we have used DS = 2 throughout this work.

### The FSCM metric

The fraction of single-copy molecules (FSCM) is a metric that to a certain extent describes how many unseen molecules that can be expected in a population of molecules. FSCM is calculated as

$$ FSCM=\frac{h(1)}{\sum_{i=1}^{\infty }h(i)} $$

where *h(i)* represents the number of molecules with *i* copies in the CU histogram.

### Retrieval of GC content and transcript length

Transcript lengths were retrieved using the GenomicFeatures [26] R package (version 1.36.4) in combination with the biomaRt [27] package (version 2.40.0). We used the biomart ENSEMBL_MART_ENSEMBL (version 103) and the dataset mmusculus_gene_ensembl (version GRCm38.p6). We calculated GC content using the R package BSgenome.Mmusculus.UCSC.mm10 [28] (version 1.4.0), in combination with GenomicFeatures and Biostrings [29].

### Correction using pooled data from other datasets

The prediction is dependent on having enough data per gene to build CU histograms, as sampling effects on the histogram leads to unstable prediction. A way to circumvent this issue is to use CU histogram data from other similar datasets in the prediction. The Preseq DS algorithm with CU histograms truncated at 2 copies per molecule is convenient in this regard, since we only need to estimate the fractions of molecules that have one (FSCM) and two (FDCM) copies. These metrics were measured per gene for the datasets PBMC_V3, PBMC_V3_2, PBMC_NG, PBMC_NG_2, PBMC_V2 and EVALPBMC (pool source datasets) and were used to predict the dataset PBMC_V3_3.

Since each dataset has a different degree of saturation (i.e., average counts per UMI), there is a need to normalize FSCM and FDCM between the dataset being predicted and the pool source datasets (see for example Additional file 1: Fig S5 C, where the dataset PBMC_V2 on average has a lower FSCM than LC). We utilized quantile (all quantiles) normalization [30] to adjust the FSCM and FDCM of the pool source datasets to be more similar that of the dataset being predicted.

The pooled FSCM and FDCM metrics for a gene are then calculated as a weighted mean of all datasets, including the dataset being predicted, where the weight is the number of UMIs for the gene per dataset. A third metric, the fraction of molecules that have more than two copies (FMCM), is calculated from FSCM and FDCM, as FMCM = 1 - FSCM - FDCM. The histogram used for prediction is simplified to these three bins and constructed using those metrics scaled with the number of UMIs of the gene in the dataset to be predicted. Prediction is then carried out as described above.

For generating Fig. 4C, where prediction is performed using downsampled data, the pool source datasets were downsampled as well to better match the dataset being predicted regarding degree of saturation. Since much data is lost during downsampling, each dataset was repeatedly downsampled 10 times and added to the data pool, providing a list of in total 60 datasets.

### Binomial downsampling

Two datasets with similar gene amplification bias but with different sequencing depth can be batch corrected by predicting one dataset to a similar sequencing depth as the other. It is a less challenging problem to predict the gene expression of the more saturated dataset given less reads than try to predict the gene expression of the less saturated dataset given more reads. A simple method is downsampling. However, downsampling includes random sampling, which introduces a sampling noise and thus gives less accurate results. An alternative method is a procedure that we have termed *binomial downsampling*.

Binomial downsampling operates on the CU histogram *h(i)* (*i* > 0) of a gene in a pool of cells (here always the full dataset), where *i* represents the observed copies per UMI for the molecules. We seek the expression of the gene at the fraction *x* (0 < x < 1) of the original reads. The probability *p(i,j)* that a molecule with *i* copies has *j* copies after a regular downsampling can be calculated as

$$ p\left(i,j\right)= dbinom\left(j,i,x\right) $$

where *dbinom(j, i, x)* is the probability density function for the binomial distribution at *j* successes, given *i* trials and the probability *x* of success in each trial. The downsampled histogram *h’(j)* can thus be calculated as

$$ h'(j)={\sum}_{i=1}^{\infty }p\left(i,j\right)\ h(i) $$

The value of interest is h’(0), since this is the number of molecules lost in the downsampling. The remaining number of molecules n for the gene is thus the sum of the non-zero part of the histogram:

$$ n={\sum}_{i=1}^{\infty }h^{\prime }(i) $$

To transform this into gene expression, the binomial downsampling is applied to all genes, followed by a CPM normalization of the data. For a gene g with a former gene expression of e_b,g_ and a downsampled gene expression of e_a,g_ (both in CPM), a scale factor *f*_*g*_ can be calculated as

$$ {f}_g=\frac{e_{a,g}}{e_{b,g}} $$

To finally correct the count matrix, each element of the gene *g* in the single-cell count matrix is then multiplied by the scale factor *f*_*g*_.

To determine the downsampling grade *x*, we selected the *x* which yielded the highest correlation (CCC) between the batch corrected datasets.

### Concordance correlation coefficient and MSE

To compare the similarity in gene expression of two samples, the Pearson correlation is not a suitable metric since it measures linear correlation, and not similarity. We instead used Lin’s concordance correlation coefficient (CCC), which describes the expected perpendicular distance from a 45° line passing through the origin [31]. We used the CCC function in the R package DescTools (version 0.99.36) to calculate the metric, using default parameters. As a complement, we also calculated the mean squared error (MSE) on the same data.

The similarity and MSE were calculated on log-transformed data, where the transformed gene expression *l*_*i*_ for gene *i* is calculated as *l*_*i*_ = *log*_2_(*e*_*i*_ + 1), where *e*_*i*_ is the gene expression of the gene in counts per million (CPM).

### Simulated data

Generation of simulated data is described in detail in Additional file 1: Supplementary Note 2. The AUC calculations were conducted using the R package pROC [32], version 1.17.0.1

### Single-cell processing

Single-cell processing for Fig. 5 was performed using Seurat [17] v. 3.1.1, following a standard workflow. For Fig. 5A, B cells were filtered keeping cells with more than 200 UMIs and a mitochondrial content of less than 15%. Ten PCs were used in the clustering and UMAP analysis. For Fig. 5C–H and Additional file 1: Fig. S24, cells were filtered keeping cells with more than 200 UMIs and a mitochondrial content of less than 10%. 10 PCs were used in the clustering and UMAP analysis.

### Figure details

In Fig. 4C, the correction is based on correcting the UMIs for all genes individually. The prediction range is the same for all genes, scaling up the counts to match the total counts in the full dataset. The predicted UMIs are then CPM normalized.

To avoid the uncertainty in the FSCM calculations that arise from having too few molecules, only genes with at least 200 molecules in both datasets were included in Fig. 2B and Additional file 1: Fig. S5. Similarly, only genes with at least 30 molecules were included in Additional file 1: Fig. S1–S3. The reason for the lower limit in this case is that a lower limit includes more genes, reducing differences between datasets with different total number of reads.

To avoid the uncertainty in prediction from lowly expressed genes, genes with a gene expression lower than 100 CPM were removed before calculating CCC in Fig. 4D, E, leaving in total 1018 genes. CPM was then recalculated based on these genes only to avoid the influence of lowly expressed genes, for which prediction is less accurate. The value of 100 was chosen since the prediction error at that number of molecules is roughly half of that of uncorrected data (Additional file 1: Fig. S7), which in turn is motivated by that the CCC value is affected by prediction errors from 2 predictions.

In Fig. 4E, the PBMC_NG_2 dataset is downsampled to 49 percent of the reads, which is the downsampling grade that maximizes the correlation between the datasets.

In Fig. 5G, H, each gene is scaled to have a total of 1000 counts across all clusters. Only clusters with more than 300 cells are included (in total 7 clusters), and only points for which there are at least 20 UMIs are shown.

In all figures where the gene expression of a dataset is used, the gene expression is calculated as the mean gene expression across all cells.

### Implementation

The ZTNB prediction was implemented in bustools with the addition of the ‘predict’ command using the same algorithm as PreSeqR [12, 13], and utilizing the c++ libraries Eigen [33], LBFGSpp [34], and CppOptimizationLibrary [35]. The count command in bustools has been extended with the option “--hist,” which generates CU histograms that serve as input to predict, together with the count matrix. In addition, UMI correction was implemented as the “umicorrect” command, utilizing the method of [24].

## Supplementary Information

**Additional file 1.** Supplementary information.

**Additional file 2: Table S1**. FSCM values per gene for 10X Chromium and Drop-Seq data, for both mouse and human.

**Additional file 3.** Review history.

## Data Availability

Means to access the datasets analyzed during the current study are listed in Additional file 1: Table S2. The source code as well as Jupyter notebooks for generating the figures is available in GitHub [36], as well as the source code for the branch of bustools used in this project [37]. Snapshots of the repositories are available in Zenodo [38]. Jupiter notebooks have not been produced for Fig. 5 and the data generation for Additional file 1: Fig. S6, S24, and S25, due to difficulties in setting up the right versions of R packages, but the code is directly available in the Github repository. All code is released under the BSD 2-clause license, except for the R file “modZTNB.R,” which is released under a GPL3 license to comply with the GPL3 license of PreseqR.
