# Supplementary material for: BUTTERFLY: addressing the pooled amplification paradox with unique molecular identifiers in single-cell RNA-seq
Source: Genome Biol. 2021 Jun 8;22:174. doi: 10.1186/s13059-021-02386-z (PMC8188791; doi:10.1186/s13059-021-02386-z)
Supplement: Supplementary file 1 — Additional file 1. Supplementary information. [file 13059_2021_2386_MOESM1_ESM.pdf]

Additional File 1

Supplementary Information

BUTTERFLY: addressing the pooled amplification paradox with unique molecular identifiers in single-cell RNA-seq

Johan Gustafsson[1,2], Jonathan Robinson[1,2,3], Jens Nielsen[1,2,4,*] and Lior Pachter[5,*]

[1] Department of Biology and Biological Engineering, Chalmers University of Technology, Kemivägen 10, Gothenburg, Sweden.

[2] Wallenberg Center for Protein Research, Chalmers University of Technology, Kemivägen 10, Gothenburg, Sweden.

[3] Department of Biology and Biological Engineering, National Bioinformatics Infrastructure Sweden, Science for Life Laboratory, Chalmers University of Technology, Kemivägen 10, SE-41258 Gothenburg, Sweden

[4] BioInnovation Institute, Ole Maaløes Vej 3, DK2200 Copenhagen N, Denmark

[5] Division of Biology and Biological Engineering & Department of Computing and Mathematical Sciences, California Institute of Technology, Pasadena, USA

* Corresponding author

Supplementary Figures

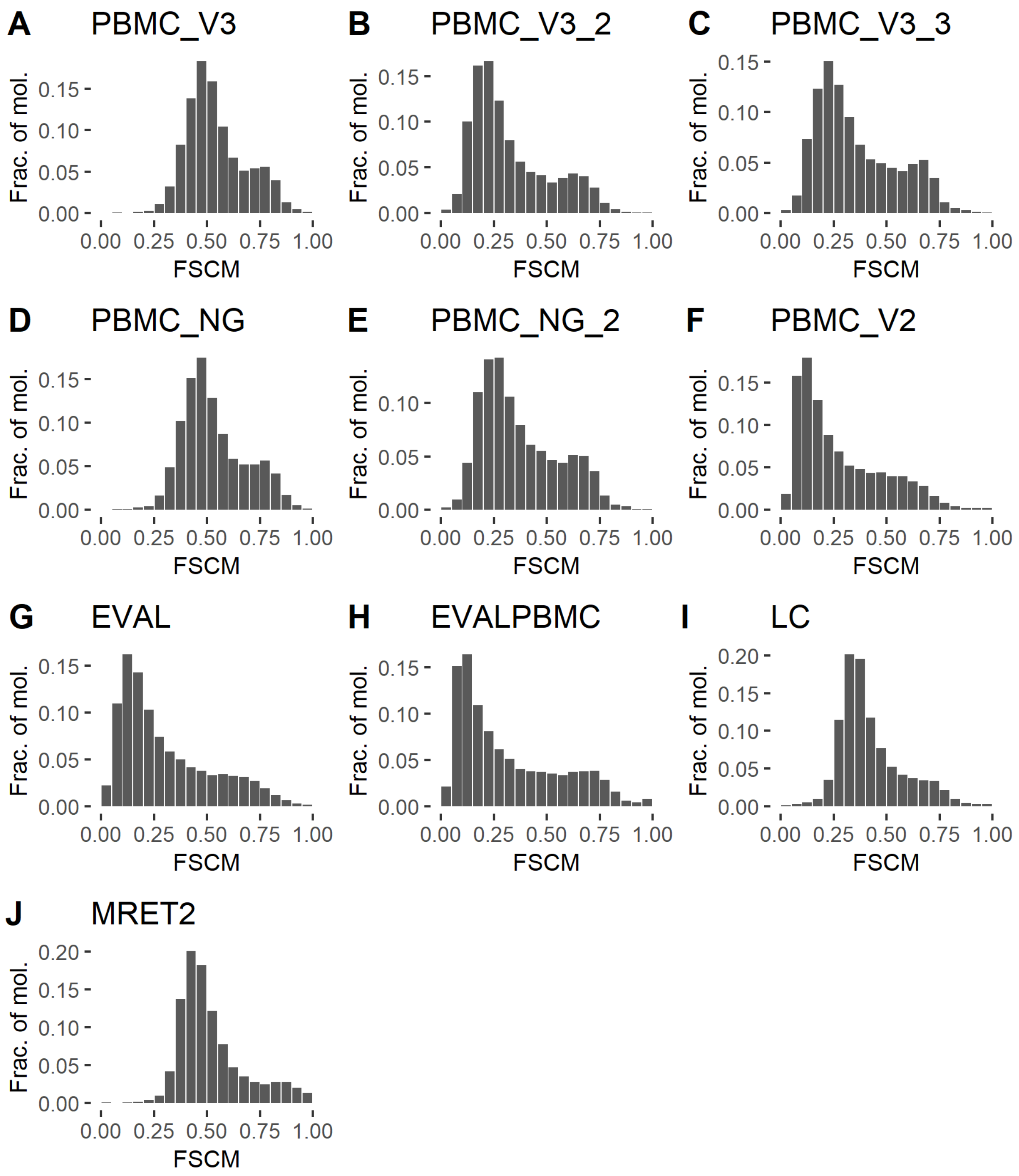

Fig S1: Histograms over fraction of single-copy molecules per gene for 10x Chromium datasets. *Genes with fewer than 30 molecules present in the dataset are not shown. The code to reproduce this figure is here: code*

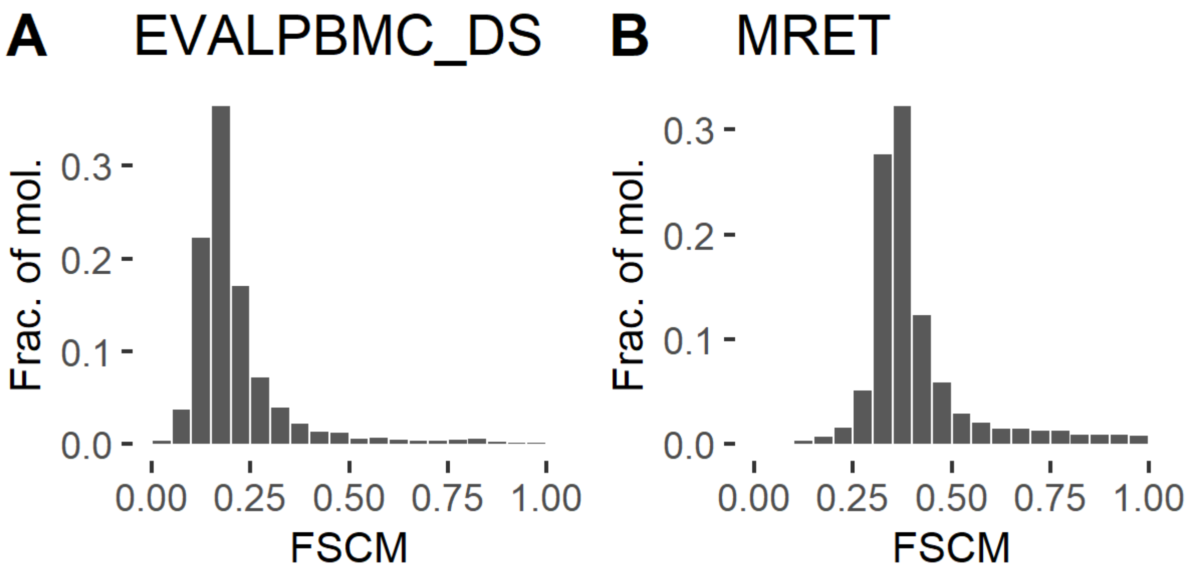

Fig S2: Histograms over fraction of single-copy molecules per gene for Drop-Seq datasets. *Genes with fewer than 30 molecules present in the dataset are not shown. The code to reproduce this figure is here: [code]*

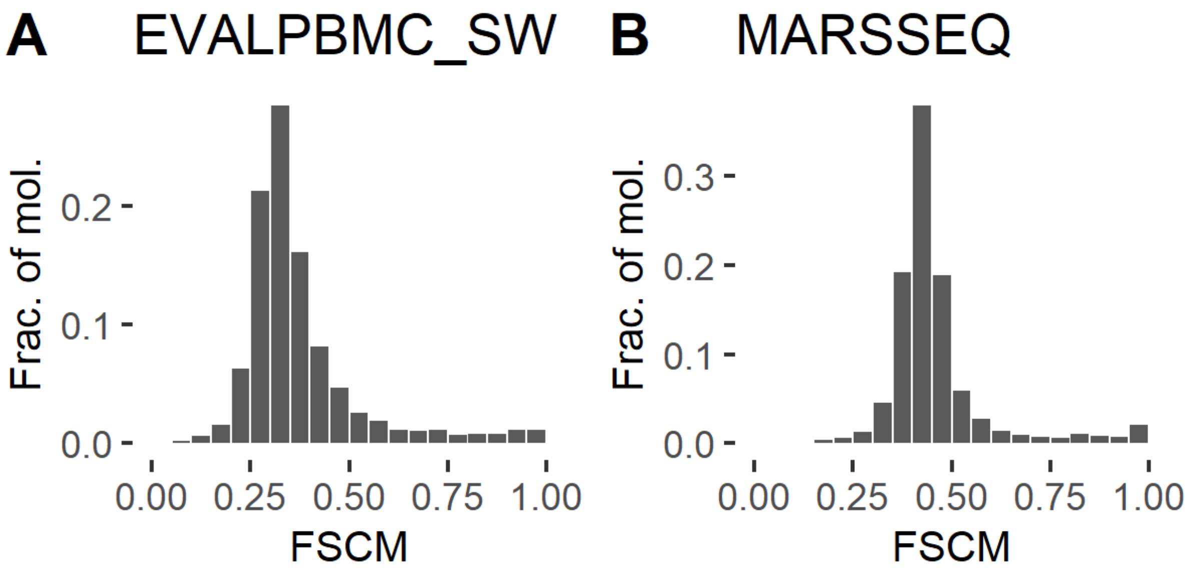

Fig S3: Histogram over fraction of single-copy molecules per gene for the Seq-Well and MARS-seq 2.0 datasets. *Genes with fewer than 30 molecules present in the dataset are not shown. The code to reproduce this figure is here: code*

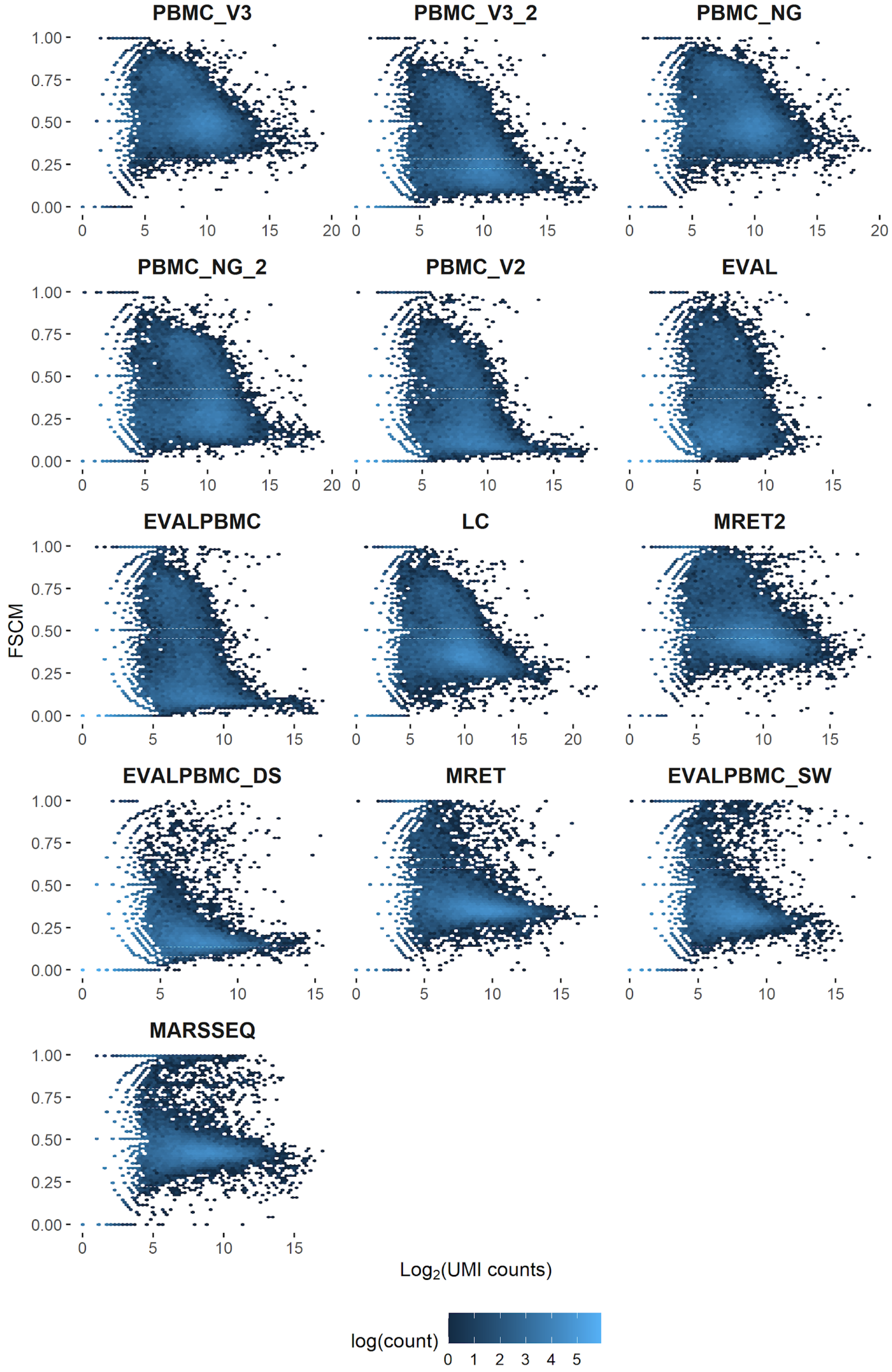

Fig S4: Fraction of single-copy molecules vs number of UMIs per gene per dataset.
The code to reproduce this figure is here: code

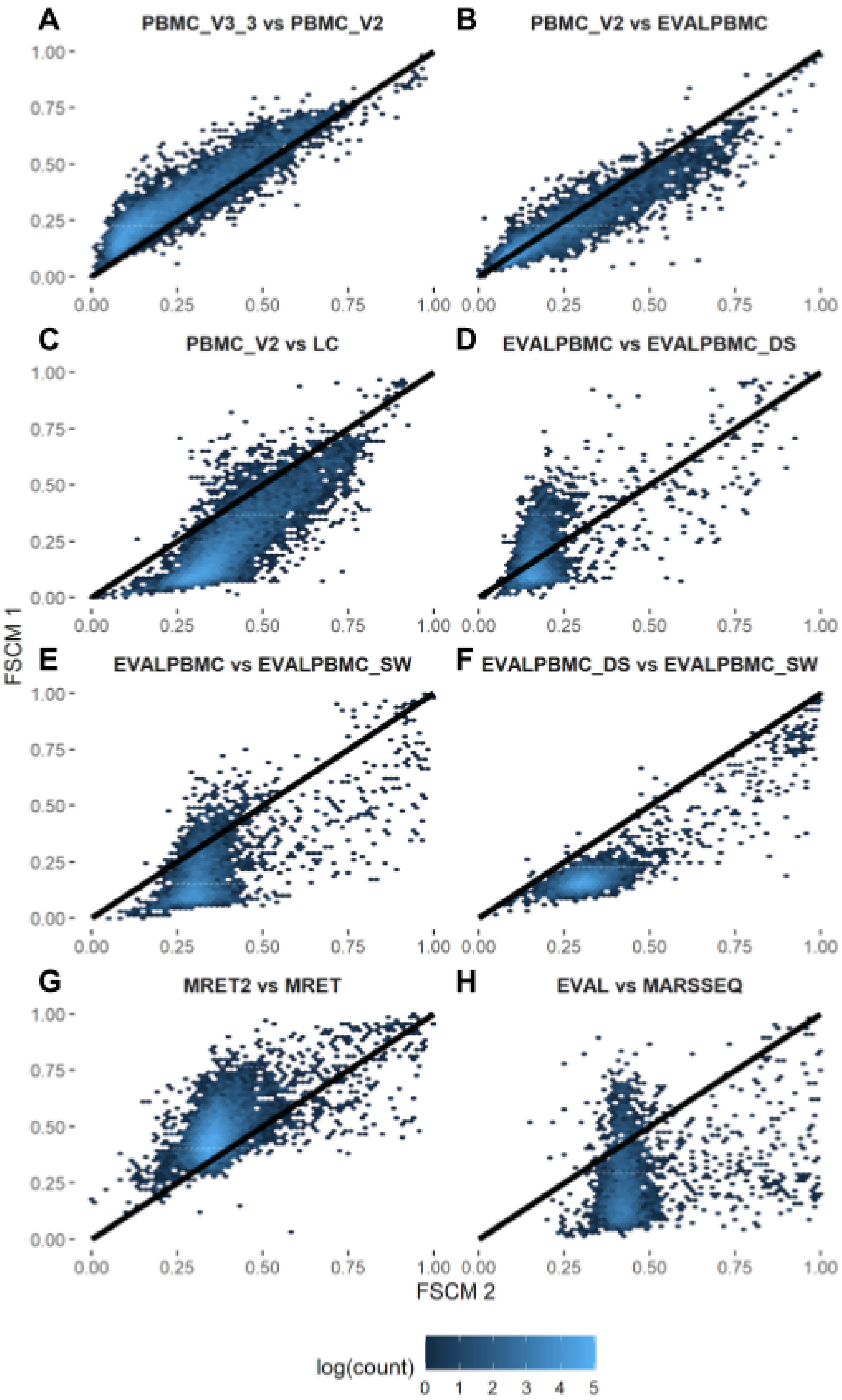
A
PBMC_V3_3 vs PBMC_V2
B
PBMC_V2 vs EVALPBMC
C
PBMC_V2 vs LC
D
EVALPBMC vs EVALPBMC_DS
E
EVALPBMC vs EVALPBMC_SW
F
EVALPBMC_DS vs EVALPBMC_SW
G
MRET2 vs MRET
H
EVAL vs MARSSEQ
FSCM 1
FSCM 2
0.00
0.25
0.50
0.75
1.00
log(count)
0
1
2
3
4
5

Fig S5: Gene-wise comparison of FSCM between pairs of datasets. *Each point represents a gene; only genes with at least 200 UMIs in both datasets are included. FSCM 1 (y axis) refers to the first dataset in the title and FSCM 2 (x axis) to the second. Note that the points are not necessarily expected to be centered around the line; the datasets compared may be differently saturated, which causes a "bent curve". A. 10x Chromium, v2 vs v3 chemistry, human PBMC. B. Both datasets from 10x Chromium, v2 chemistry, human PBMC, but from different labs. C. Both datasets from 10x Chromium, v2 chemistry, but from different labs and different tissue - human PBMC vs human lung tumor. D. 10x Chromium, v2 chemistry, vs Drop-Seq, both human PBMC, generated from the same sample. E. 10x Chromium, v2 chemistry, vs Seq-Well, both human PBMC, generated from the same sample. F. Drop-Seq vs Seq-Well, both human PBMC, generated from the same sample. G. 10x Chromium, v2 chemistry, vs Drop-Seq, both mouse retina but generated by different research groups. H. 10x Chromium, v2 chemistry, vs MARS-Seq 2.0, both from different labs and different tissue - mouse brain vs mouse ES cells and mouse embryonic fibroblasts.*
The code to reproduce this figure is here: code

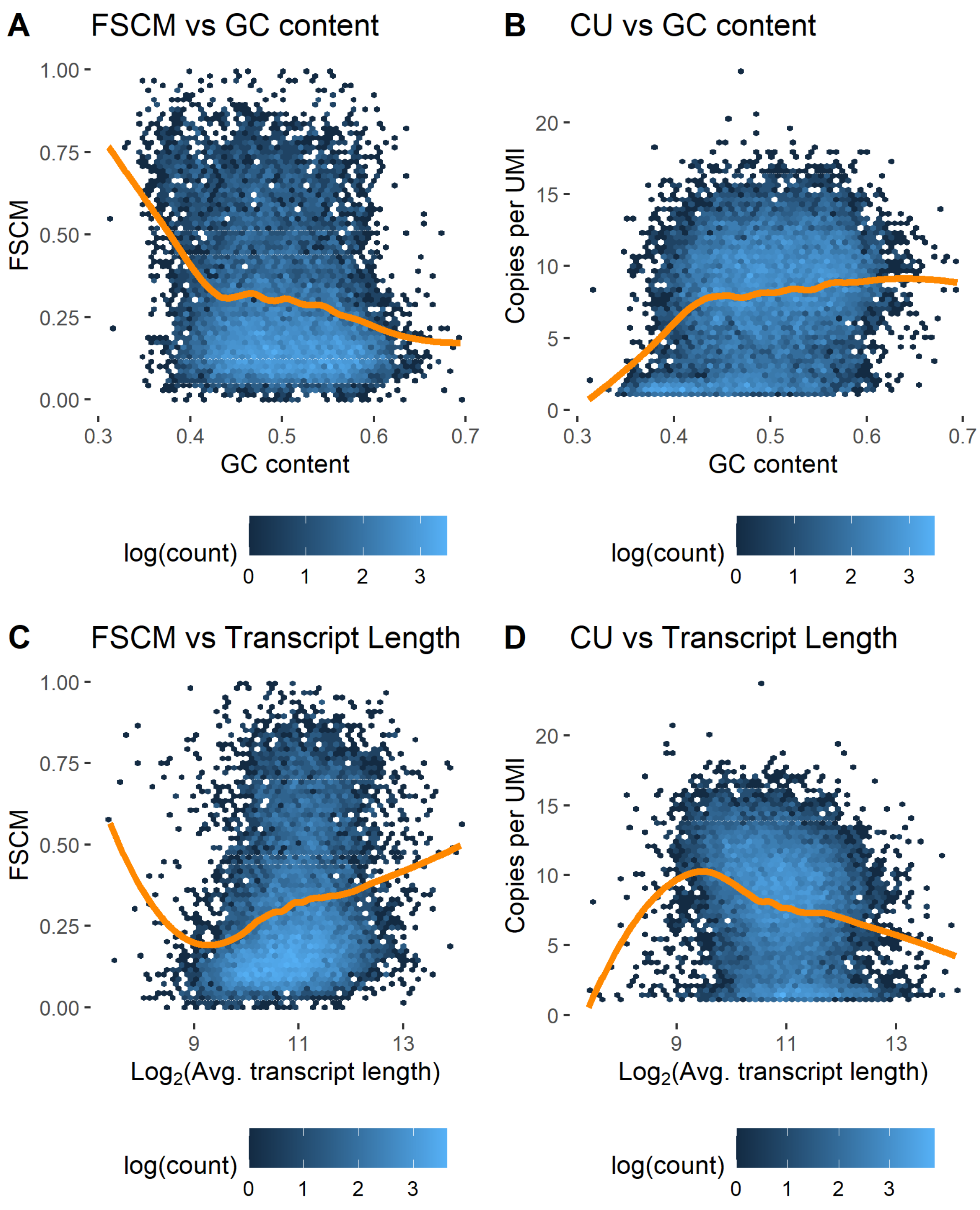

Fig S6: Comparison of amplification per gene with transcript length and GC content. *GC content and transcript length are calculated from the mouse genome, where the values represent the average over all transcripts for a gene. The fraction of single-copy molecules (FSCM) and copies per UMI (CU) metrics are measured in the EVAL dataset. Each point represents a gene; only genes with at least 30 UMIs in the EVAL dataset are included. The code to reproduce this figure is here: code*

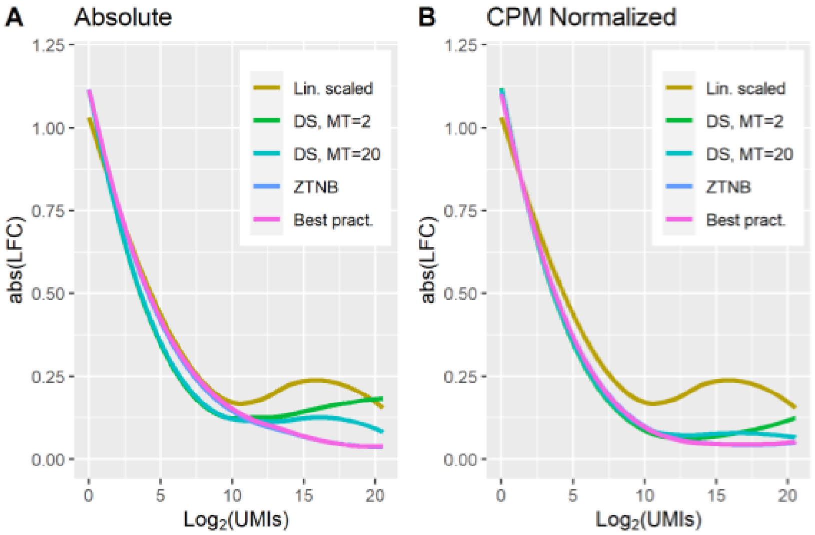

Method	CCC
Lin. Scaled	0.9816
DS, MT=2	0.9904
DS, MT=20	0.9900
ZTNB	0.9897
Best pract.	0.9898

Fig S7: Prediction errors for different methods. *Each curve represents a loess fit of abs(Log$_2$ Fold Change) over all genes and all datasets, where the predicted value is compared to ground truth. A. Direct comparison of predicted molecules per gene. B. Comparison of predicted molecules per gene after CPM normalization. The normalization removes systematic errors in prediction across genes, for example if a method underestimates all predictions. The CCC values presented are calculated on log-transformed CPM-normalized data. The code to reproduce this figure is here: code*

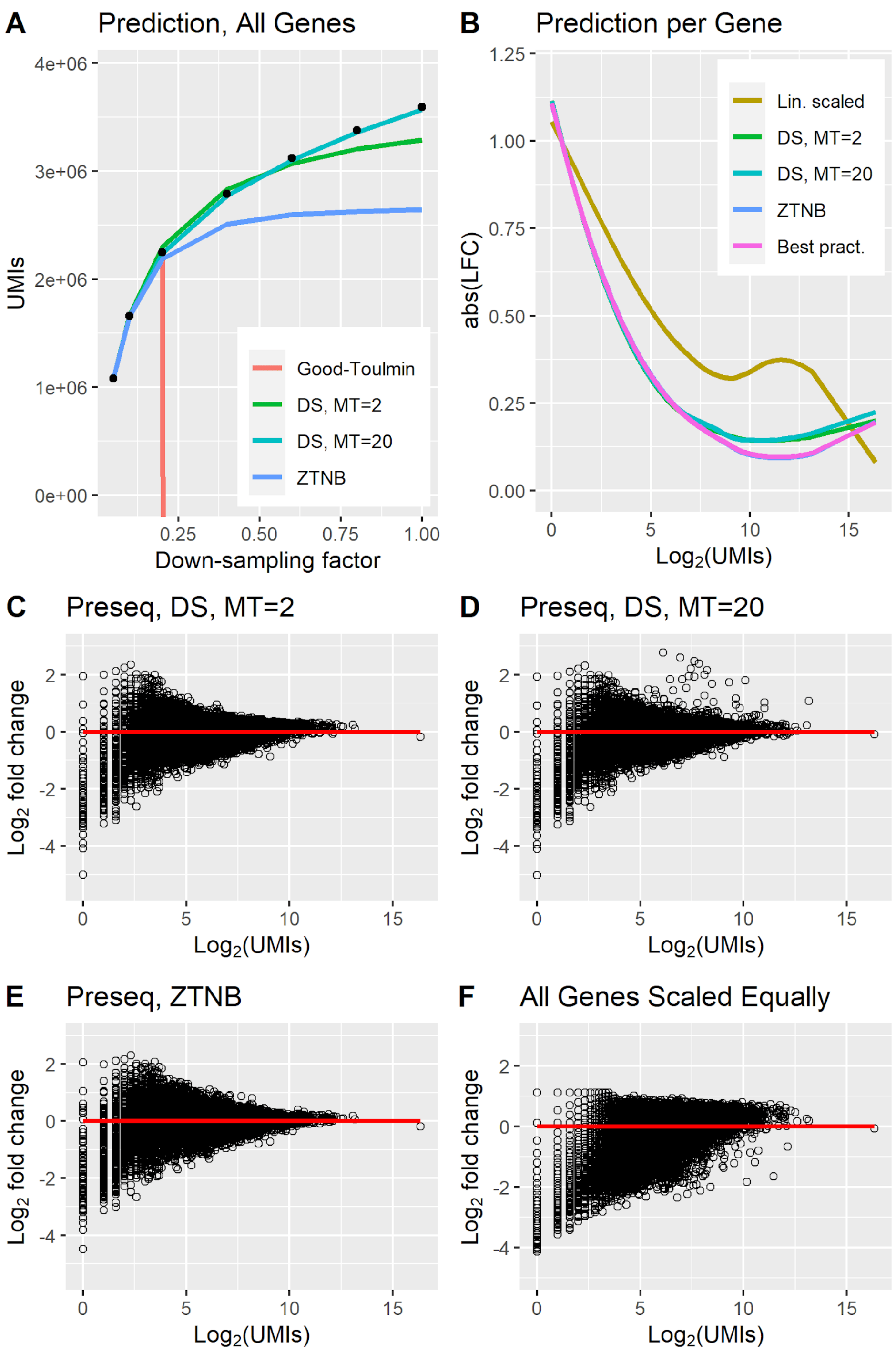

Fig S8: Correction evaluation for the EVAL dataset. *The data was downsampled to 1/20 for A and to 1/10 for B-F, and the corrected expression using different prediction methods was compared to ground truth (predicting to 10 times the number of counts for B-F). A. Prediction of all UMIs in the dataset, collected into a single pool. The data was corrected from 1/20 of the reads. Ground truth is represented by black dots. B. Correction errors for different prediction methods on CPM-normalized data. The figure shows a loess fit of abs(LFC) over all genes. C-F. Scatter plots showing the correction error for each gene as the Log_2 fold change between corrected expression and ground truth (CPM normalized). The x axis corresponds to Log_2 of the number of UMIs for the gene in the downsampled data. The code to reproduce this figure is here: code*

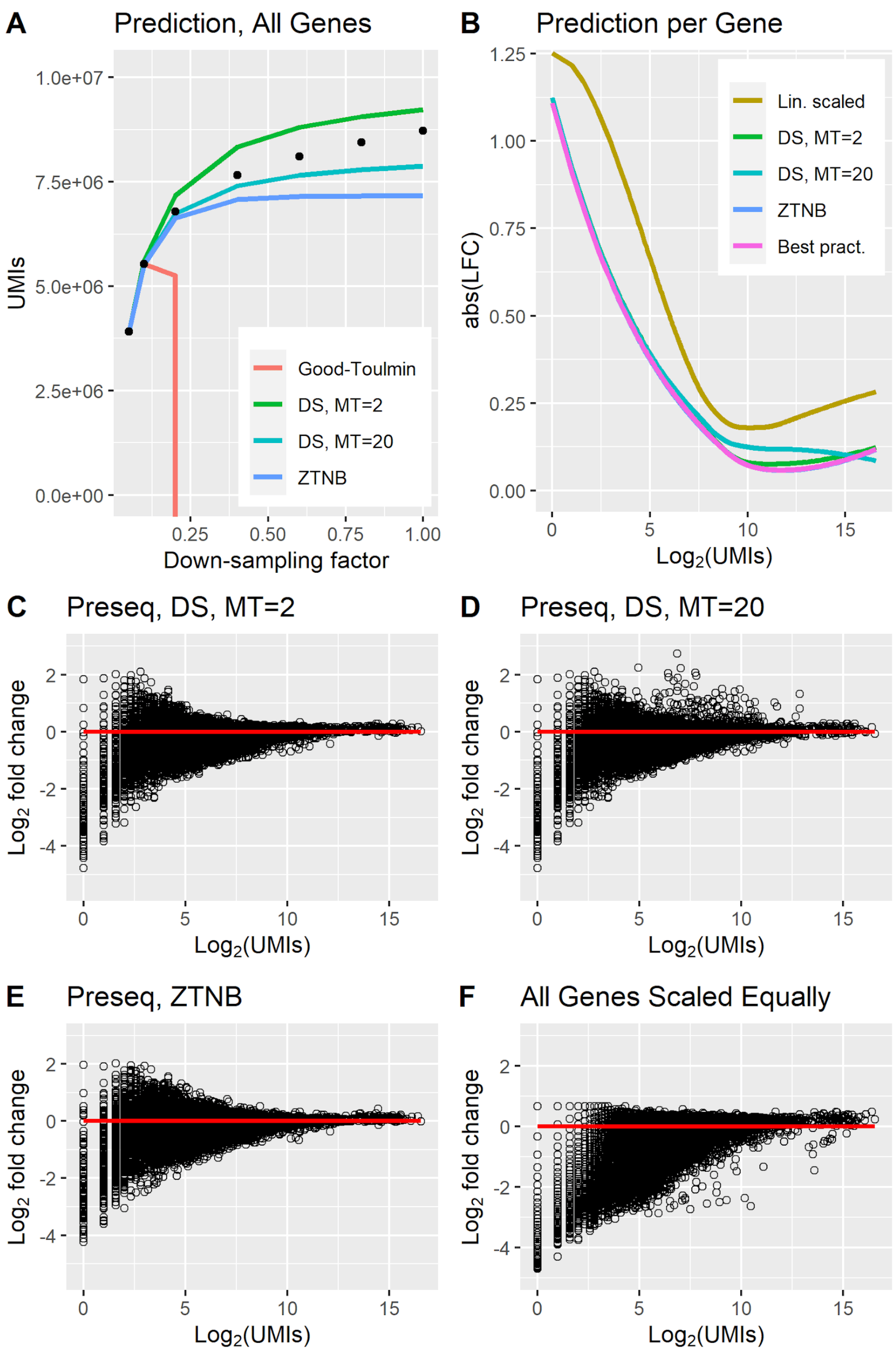

Fig S9: Correction evaluation for the EVALPBMC dataset. *The data was downsampled to 1/20 for A and to 1/10 for B-F, and the corrected expression using different prediction methods was compared to ground truth (predicting to 10 times the number of counts for B-F). A. Prediction of all UMIs in the dataset, collected into a single pool. The data was corrected from 1/20 of the reads. Ground truth is represented by black dots. B. Correction errors for different prediction methods on CPM-normalized data. The figure shows a loess fit of abs(LFC) over all genes. C-F. Scatter plots showing the correction error for each gene as the* Log_2 *fold change between corrected expression and ground truth (CPM normalized). The x axis corresponds to* Log_2 *of the number of UMIs for the gene in the downsampled data. The code to reproduce this figure is here: code*

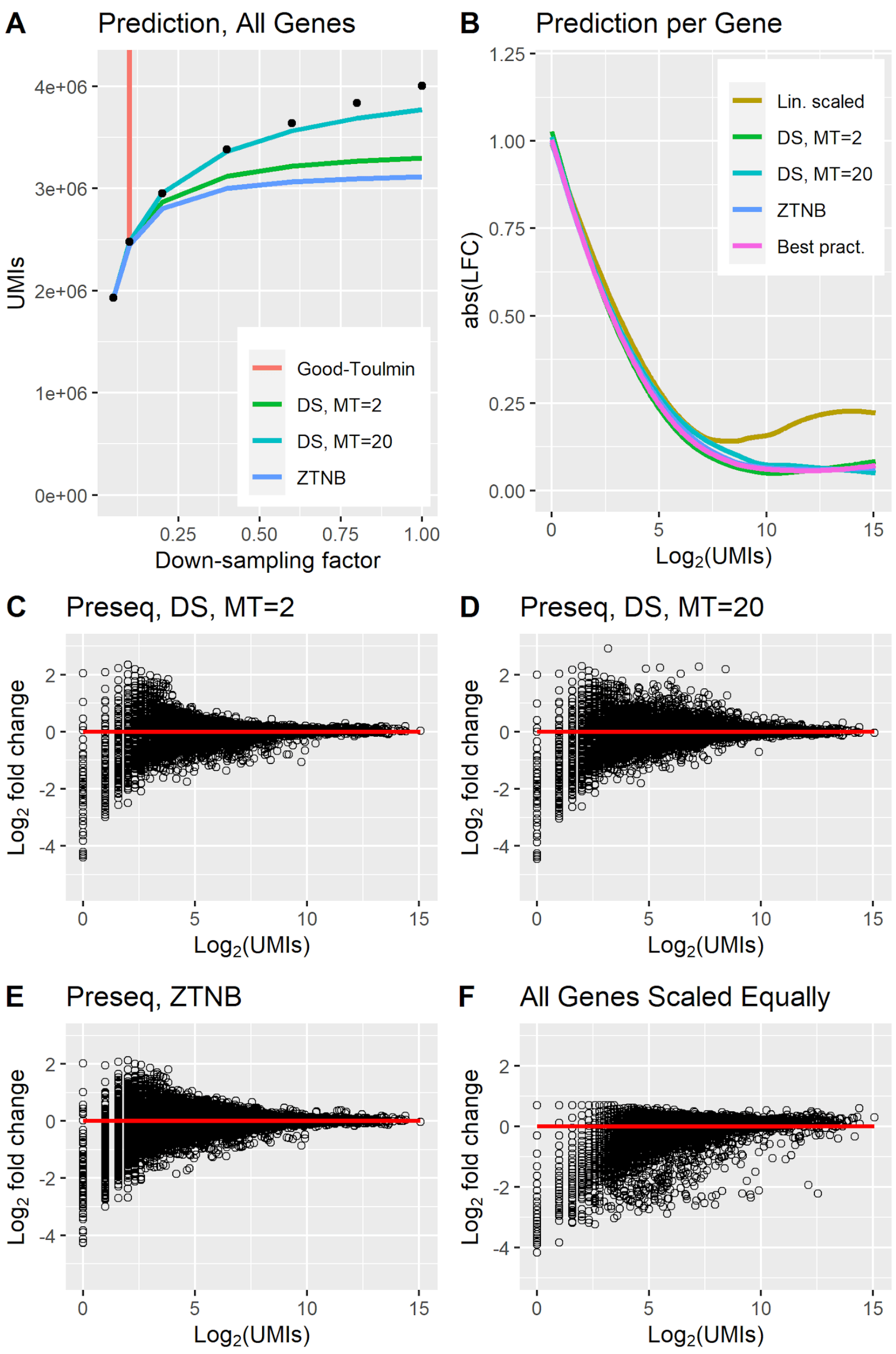

Fig S10: Correction evaluation for the EVALPBMC_DS dataset. *The data was downsampled to 1/20 for A and to 1/10 for B-F, and the corrected expression using different prediction methods was compared to ground truth (predicting to 10 times the number of counts for B-F). A. Prediction of all UMIs in the dataset, collected into a single pool. The data was corrected from 1/20 of the reads. Ground truth is represented by black dots. B. Correction errors for different prediction methods on CPM-normalized data. The figure shows a loess fit of abs(LFC) over all genes. C-F. Scatter plots showing the correction error for each gene as the Log_2 fold change between corrected expression and ground truth (CPM normalized). The x axis corresponds to Log_2 of the number of UMIs for the gene in the downsampled data. The code to reproduce this figure is here: code*

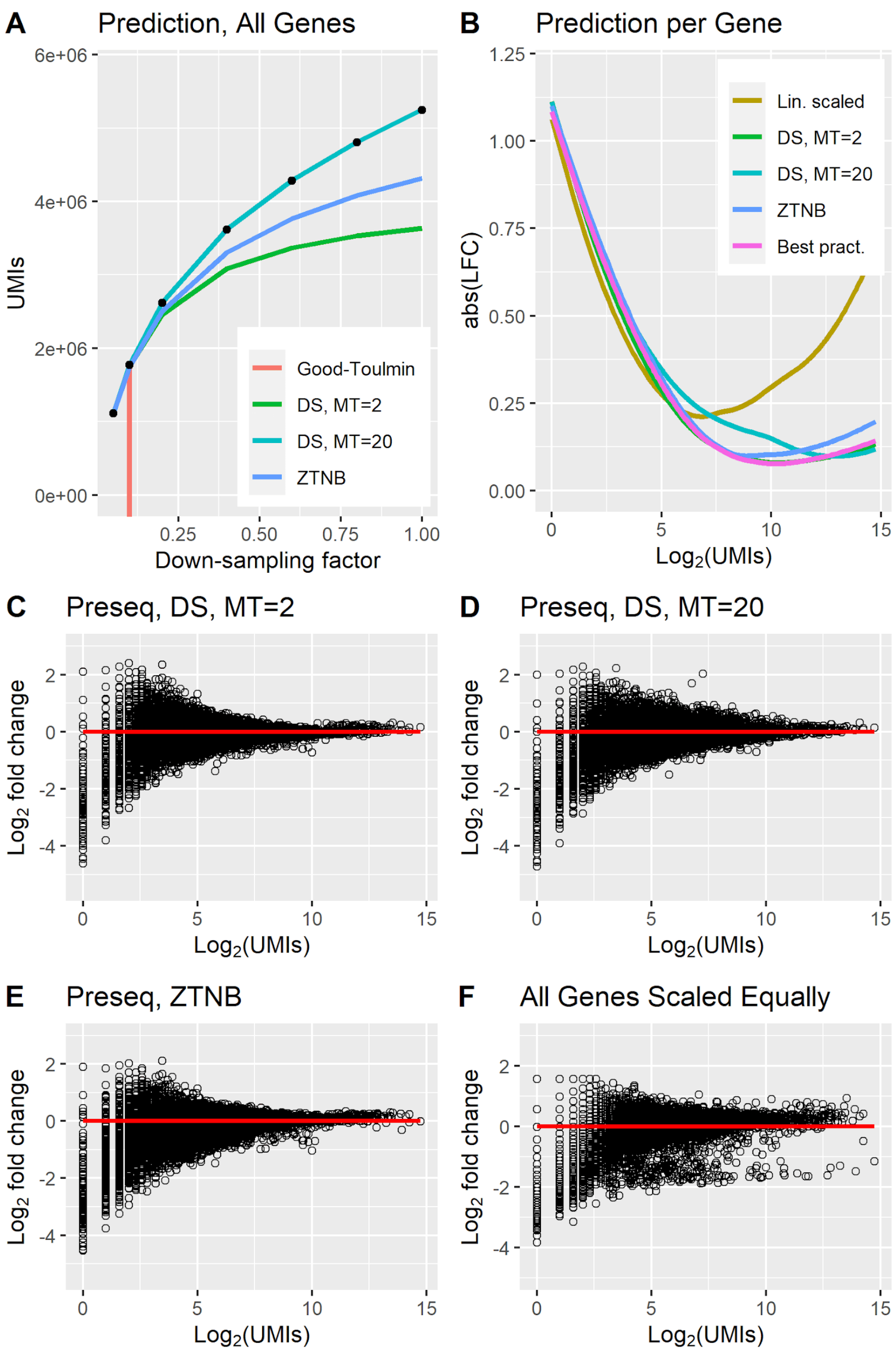

Fig S11: Correction evaluation for the EVALPBMC_SW dataset. *The data was downsampled to 1/20 for A and to 1/10 for B-F, and the corrected expression using different prediction methods was compared to ground truth (predicting to 10 times the number of counts for B-F). A. Prediction of all UMIs in the dataset, collected into a single pool. The data was corrected from 1/20 of the reads. Ground truth is represented by black dots. B. Correction errors for different prediction methods on CPM-normalized data. The figure shows a loess fit of abs(LFC) over all genes. C-F. Scatter plots showing the correction error for each gene as the Log_2 fold change between corrected expression and ground truth (CPM normalized). The x axis corresponds to Log_2 of the number of UMIs for the gene in the downsampled data. The code to reproduce this figure is here: code*

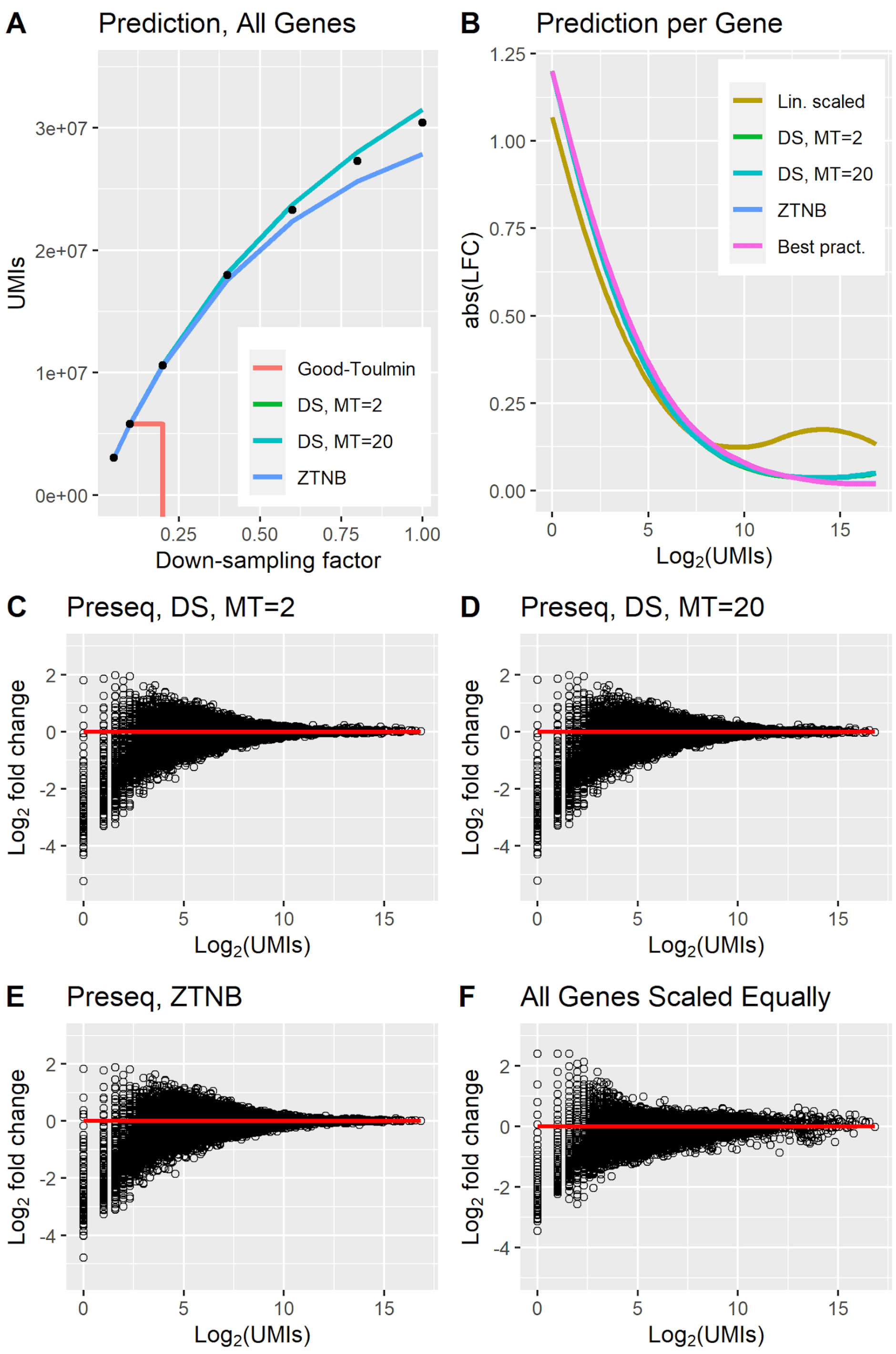

Fig S12: Correction evaluation for the PBMC_V3 dataset. *The data was downsampled to 1/20 for A and to 1/10 for B-F, and the corrected expression using different prediction methods was compared to ground truth (predicting to 10 times the number of counts for B-F). A. Prediction of all UMIs in the dataset, collected into a single pool. The data was corrected from 1/20 of the reads. Ground truth is represented by black dots. B. Correction errors for different prediction methods on CPM-normalized data. The figure shows a loess fit of abs(LFC) over all genes. C-F. Scatter plots showing the correction error for each gene as the Log_2 fold change between corrected expression and ground truth (CPM normalized). The x axis corresponds to Log_2 of the number of UMIs for the gene in the downsampled data. The code to reproduce this figure is here: [code]*

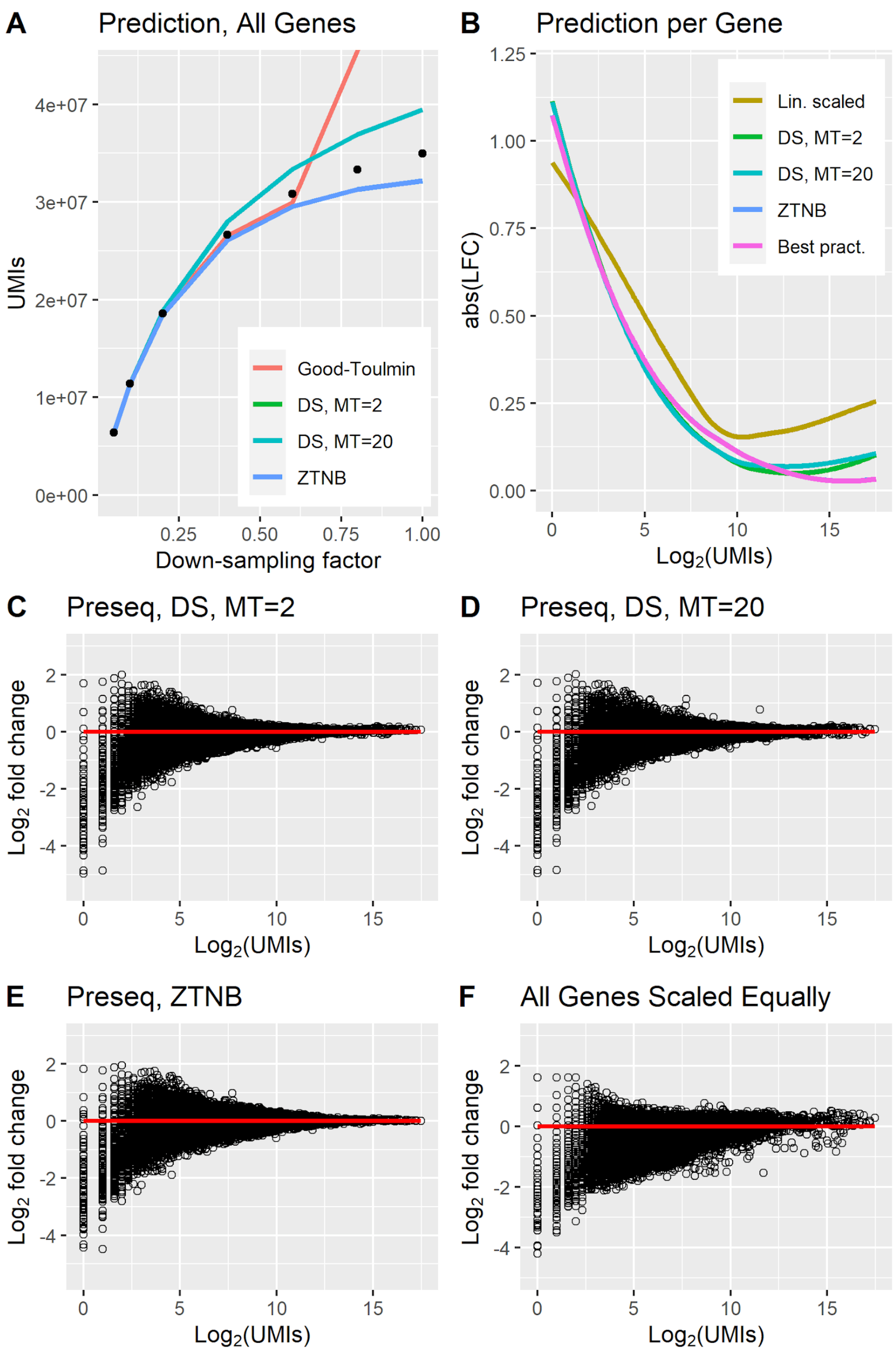

Fig S13: Correction evaluation for the PBMC_V3_2 dataset. *The data was downsampled to 1/20 for A and to 1/10 for B-F, and the corrected expression using different prediction methods was compared to ground truth (predicting to 10 times the number of counts for B-F). A. Prediction of all UMIs in the dataset, collected into a single pool. The data was corrected from 1/20 of the reads. Ground truth is represented by black dots. B. Correction errors for different prediction methods on CPM-normalized data. The figure shows a loess fit of abs(LFC) over all genes. C-F. Scatter plots showing the correction error for each gene as the Log_2 fold change between corrected expression and ground truth (CPM normalized). The x axis corresponds to Log_2 of the number of UMIs for the gene in the downsampled data. The code to reproduce this figure is here: [code]*

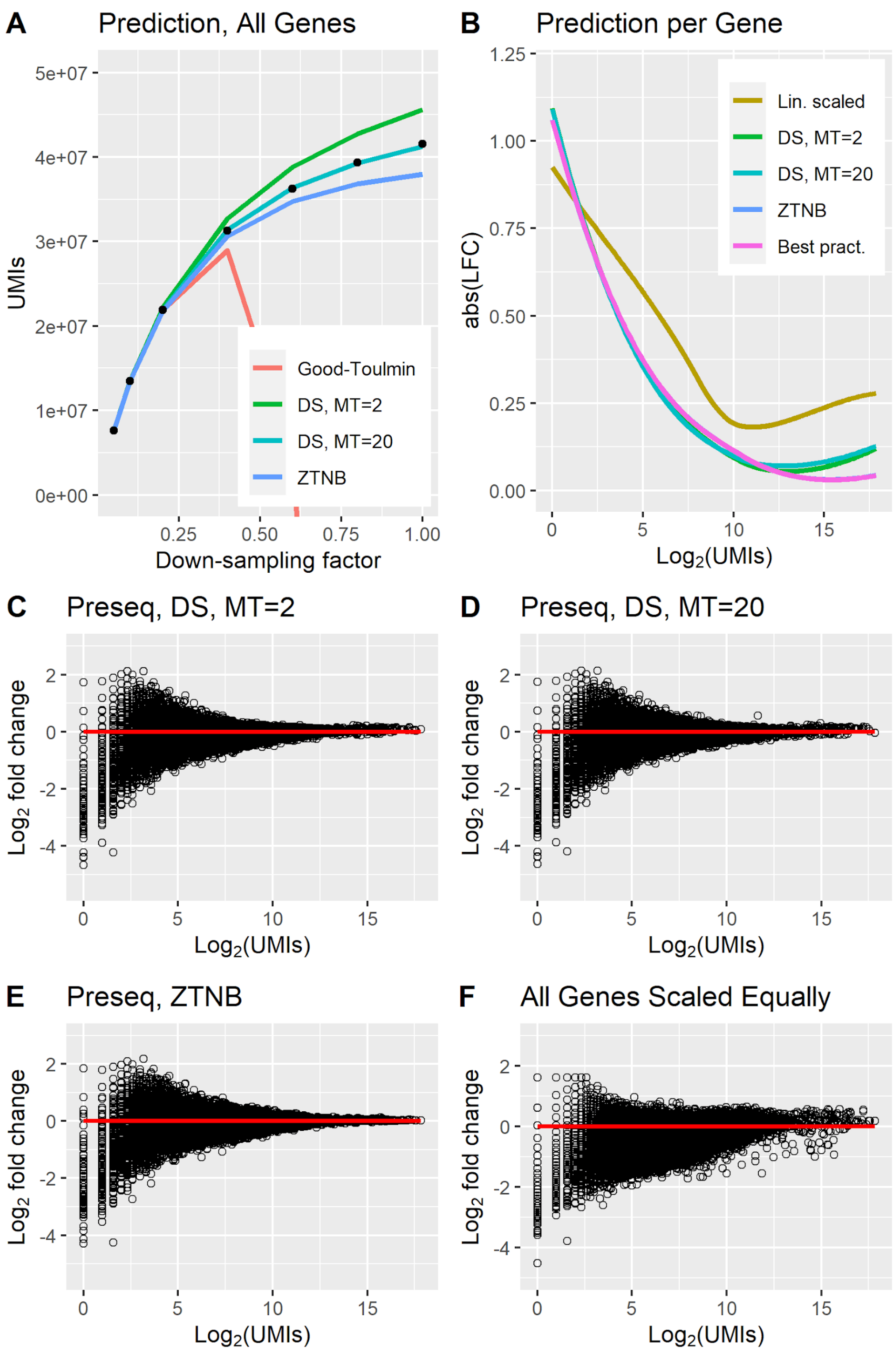

Fig S14: Correction evaluation for the PBMC_V3_3 dataset. *The data was downsampled to 1/20 for A and to 1/10 for B-F, and the corrected expression using different prediction methods was compared to ground truth (predicting to 10 times the number of counts for B-F). A. Prediction of all UMIs in the dataset, collected into a single pool. The data was corrected from 1/20 of the reads. Ground truth is represented by black dots. B. Correction errors for different prediction methods on CPM-normalized data. The figure shows a loess fit of abs(LFC) over all genes. C-F. Scatter plots showing the correction error for each gene as the Log_2 fold change between corrected expression and ground truth (CPM normalized). The x axis corresponds to Log_2 of the number of UMIs for the gene in the downsampled data. The code to reproduce this figure is here: code*

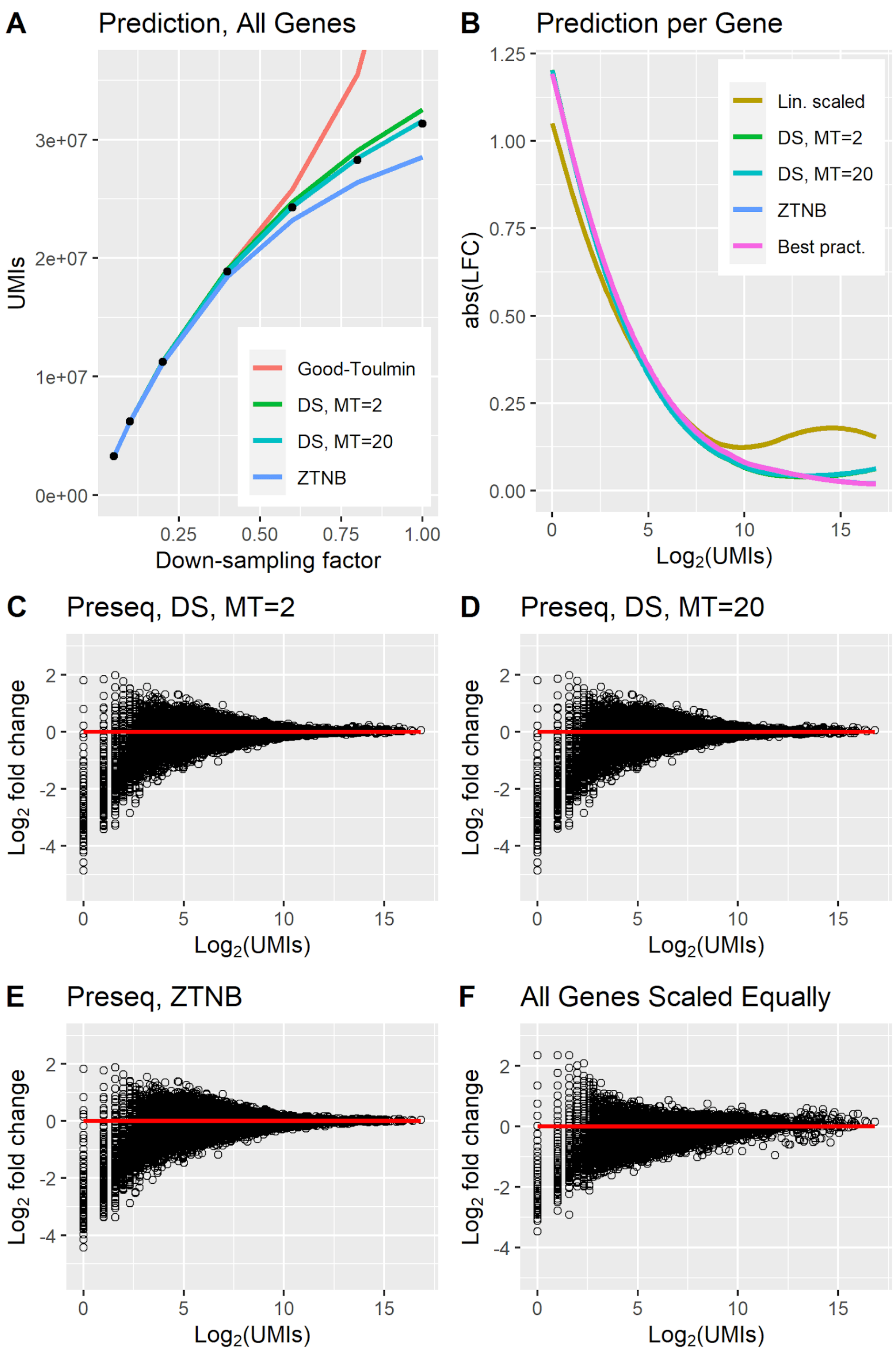

Fig S15: Correction evaluation for the PBMC_NG dataset. *The data was downsampled to 1/20 for A and to 1/10 for B-F, and the corrected expression using different prediction methods was compared to ground truth (predicting to 10 times the number of counts for B-F). A. Prediction of all UMIs in the dataset, collected into a single pool. The data was corrected from 1/20 of the reads. Ground truth is represented by black dots. B. Correction errors for different prediction methods on CPM-normalized data. The figure shows a loess fit of abs(LFC) over all genes. C-F. Scatter plots showing the correction error for each gene as the* Log_2 *fold change between corrected expression and ground truth (CPM normalized). The x axis corresponds to* Log_2 *of the number of UMIs for the gene in the downsampled data. The code to reproduce this figure is here: code*

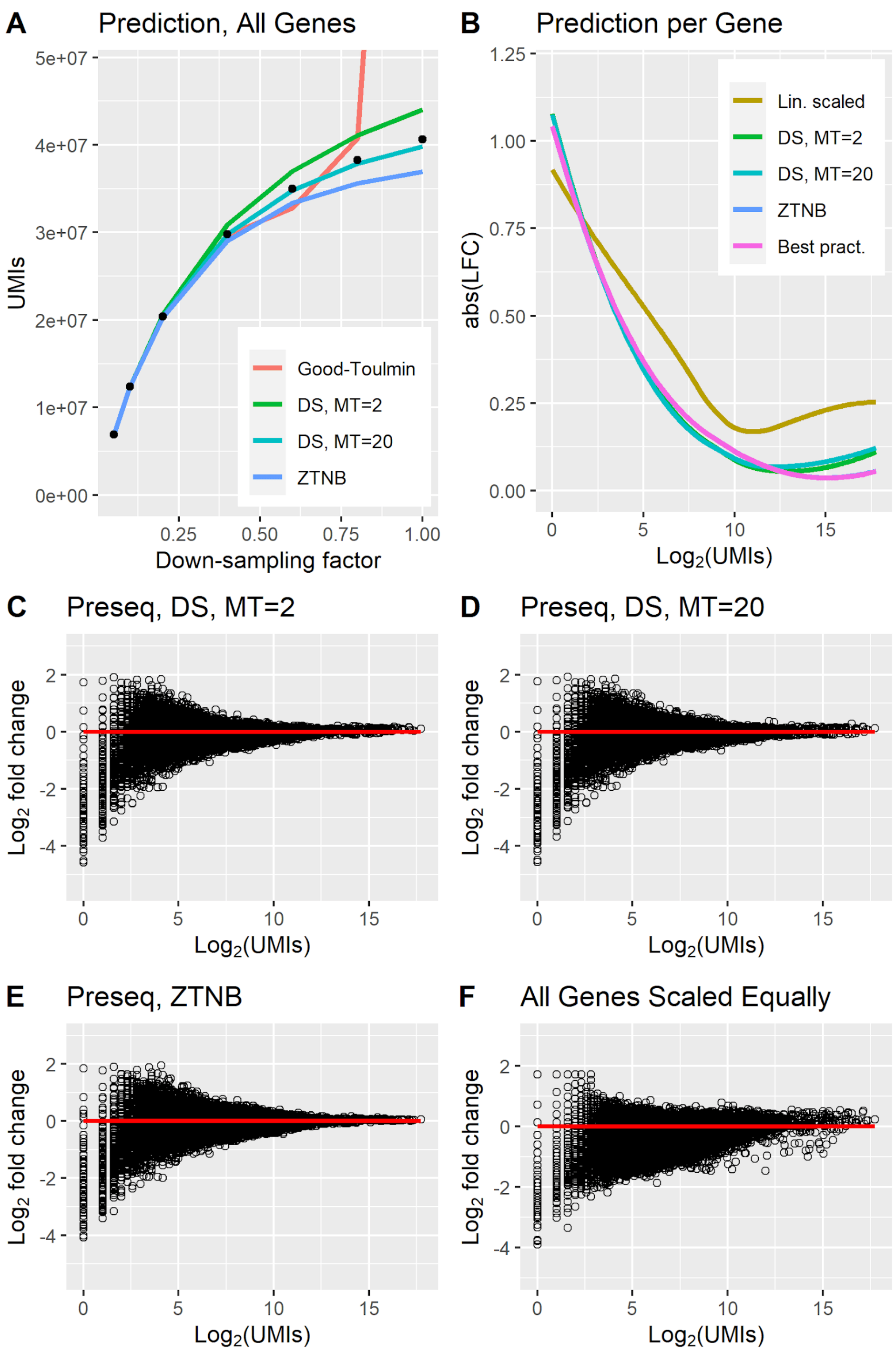

Fig S16: Correction evaluation for the PBMC_NG_2 dataset. *The data was downsampled to 1/20 for A and to 1/10 for B-F, and the corrected expression using different prediction methods was compared to ground truth (predicting to 10 times the number of counts for B-F). A. Prediction of all UMIs in the dataset, collected into a single pool. The data was corrected from 1/20 of the reads. Ground truth is represented by black dots. B. Correction errors for different prediction methods on CPM-normalized data. The figure shows a loess fit of abs(LFC) over all genes. C-F. Scatter plots showing the correction error for each gene as the Log_2 fold change between corrected expression and ground truth (CPM normalized). The x axis corresponds to Log_2 of the number of UMIs for the gene in the downsampled data. The code to reproduce this figure is here: code*

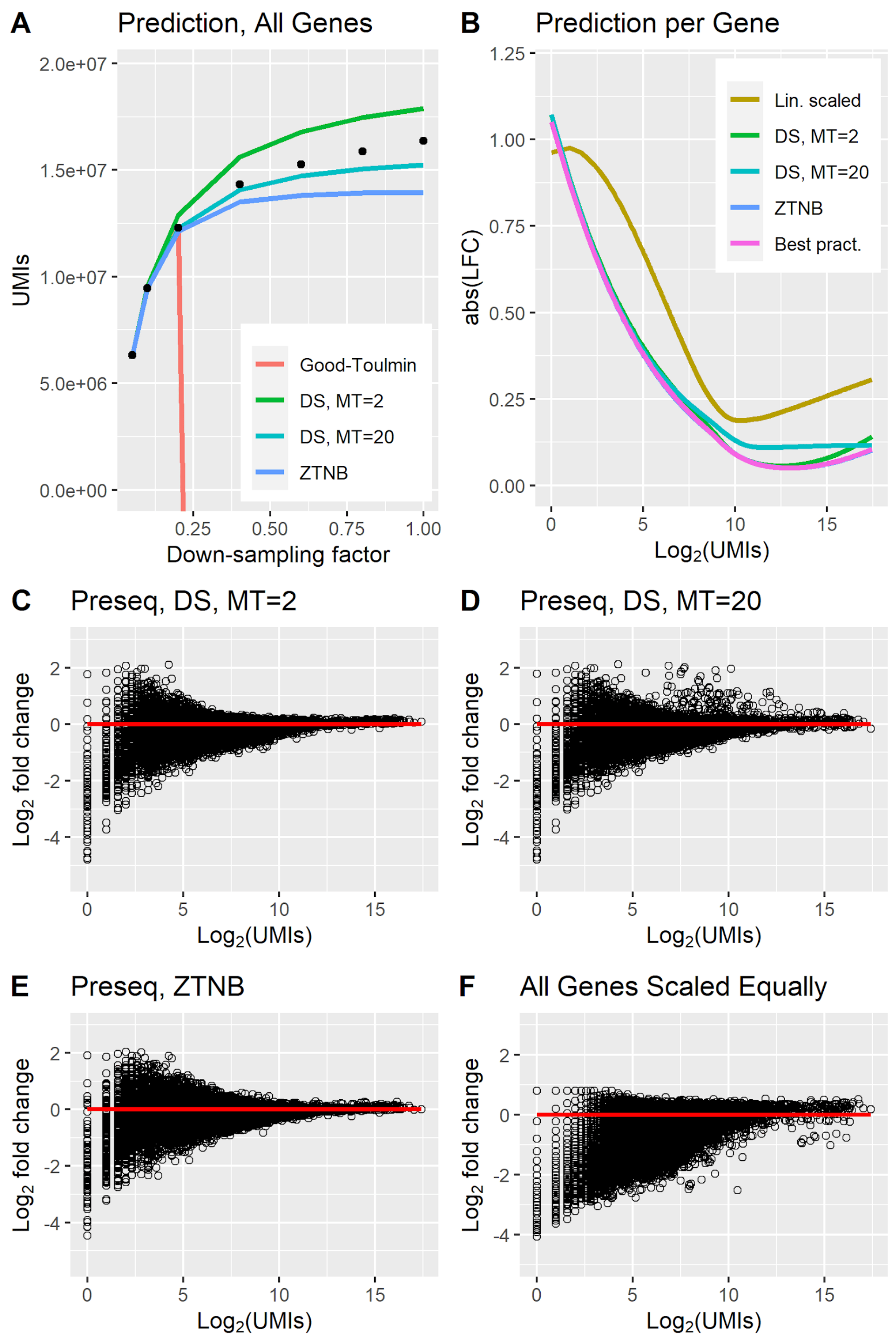

Fig S17: Correction evaluation for the PBMC_V2 dataset. *The data was downsampled to 1/20 for A and to 1/10 for B-F, and the corrected expression using different prediction methods was compared to ground truth (predicting to 10 times the number of counts for B-F). A. Prediction of all UMIs in the dataset, collected into a single pool. The data was corrected from 1/20 of the reads. Ground truth is represented by black dots. B. Correction errors for different prediction methods on CPM-normalized data. The figure shows a loess fit of abs(LFC) over all genes. C-F. Scatter plots showing the correction error for each gene as the* Log_2 *fold change between corrected expression and ground truth (CPM normalized). The x axis corresponds to* Log_2 *of the number of UMIs for the gene in the downsampled data. The code to reproduce this figure is here: code*

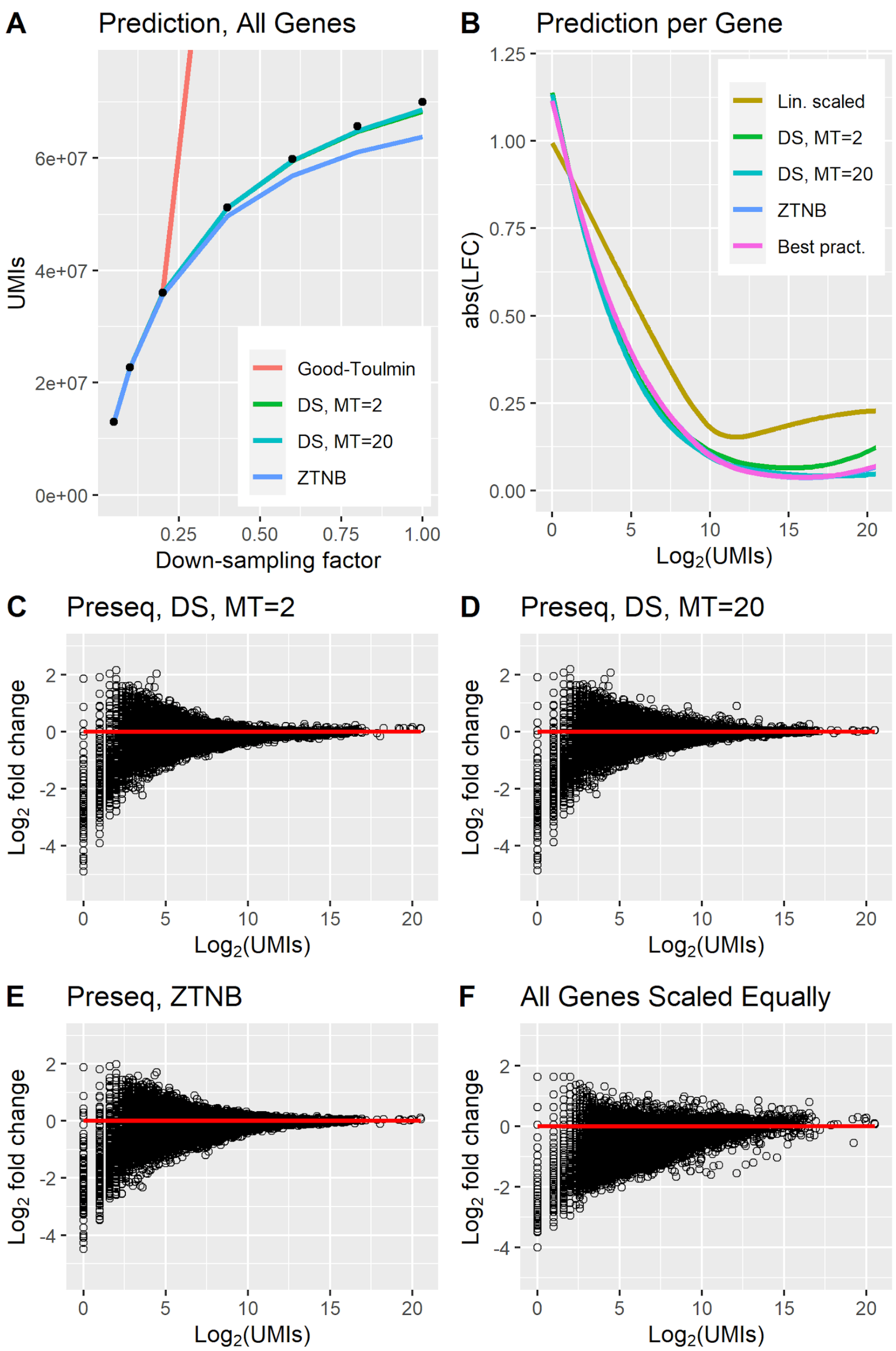

Fig S18: Correction evaluation for the LC dataset. *The data was downsampled to 1/20 for A and to 1/10 for B-F, and the corrected expression using different prediction methods was compared to ground truth (predicting to 10 times the number of counts for B-F). A. Prediction of all UMIs in the dataset, collected into a single pool. The data was corrected from 1/20 of the reads. Ground truth is represented by black dots. B. Correction errors for different prediction methods on CPM-normalized data. The figure shows a loess fit of abs(LFC) over all genes. C-F. Scatter plots showing the correction error for each gene as the Log_2 fold change between corrected expression and ground truth (CPM normalized). The x axis corresponds to Log_2 of the number of UMIs for the gene in the downsampled data.*
The code to reproduce this figure is here: [code]

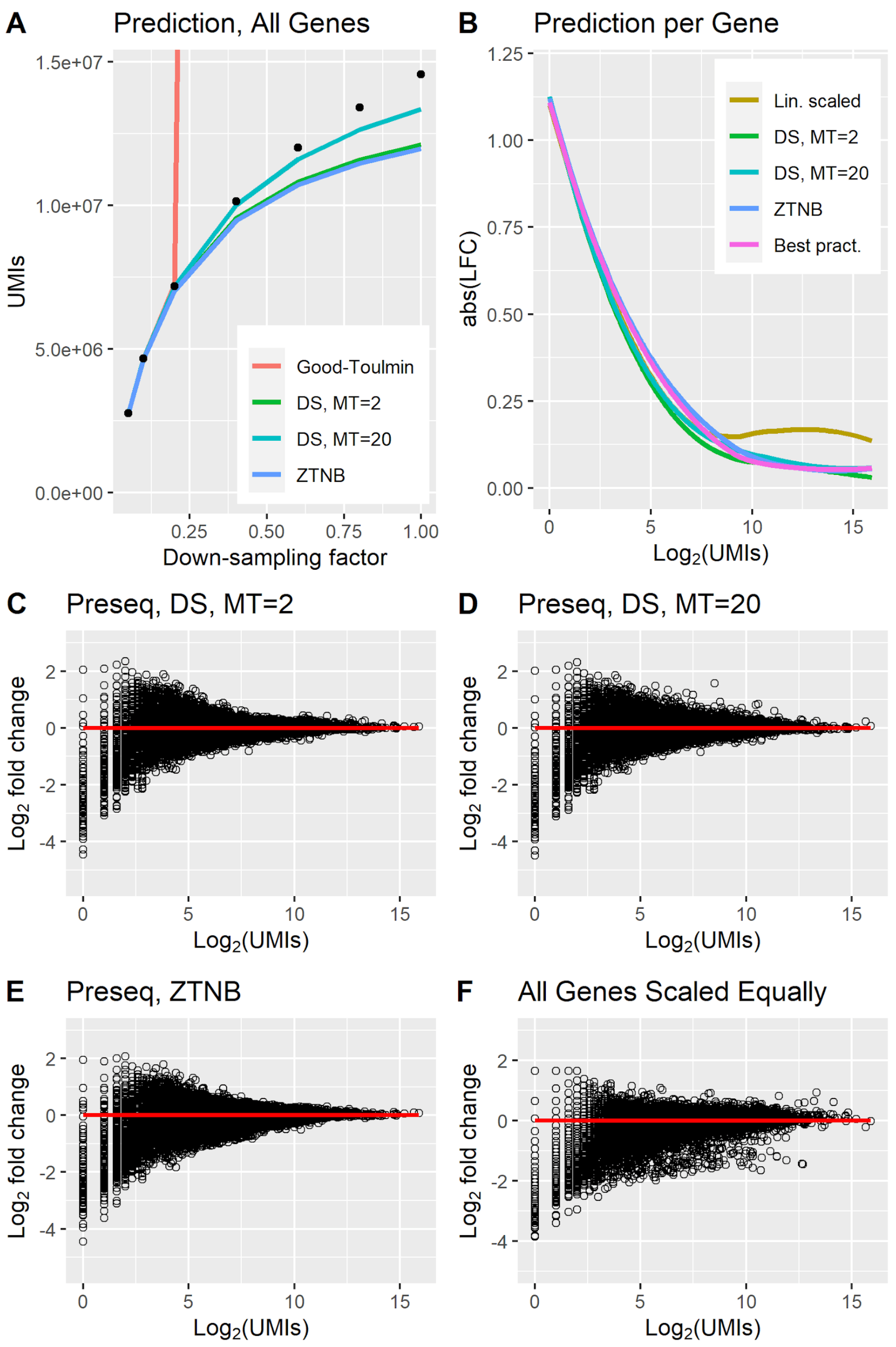

Fig S19: Correction evaluation for the MRET dataset. *The data was downsampled to 1/20 for A and to 1/10 for B-F, and the corrected expression using different prediction methods was compared to ground truth (predicting to 10 times the number of counts for B-F). A. Prediction of all UMIs in the dataset, collected into a single pool. The data was corrected from 1/20 of the reads. Ground truth is represented by black dots. B. Correction errors for different prediction methods on CPM-normalized data. The figure shows a loess fit of abs(LFC) over all genes. C-F. Scatter plots showing the correction error for each gene as the Log_2 fold change between corrected expression and ground truth (CPM normalized). The x axis corresponds to Log_2 of the number of UMIs for the gene in the downsampled data. The code to reproduce this figure is here: [code]*

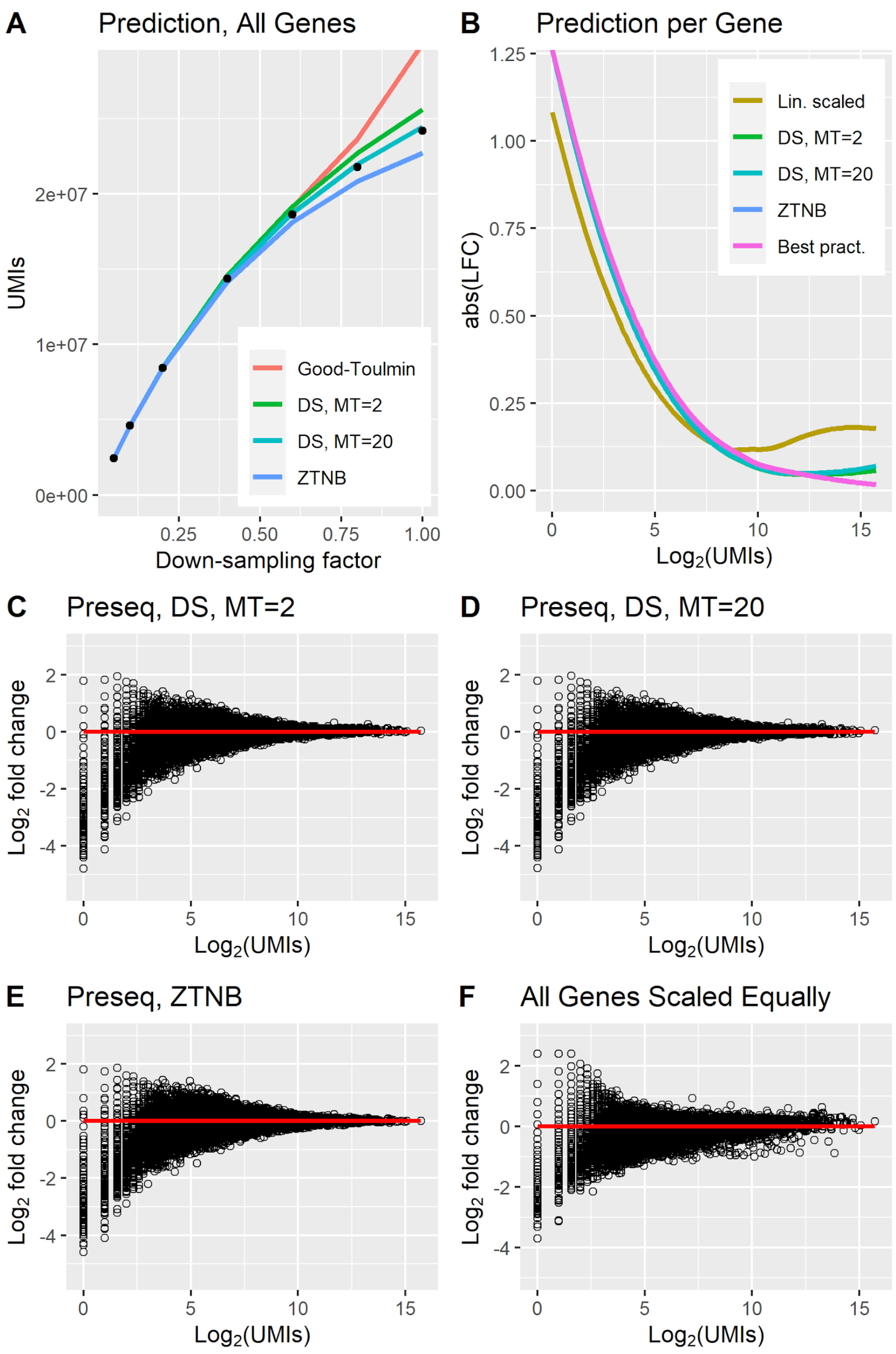

Fig S20: Correction evaluation for the MRET2 dataset. *The data was downsampled to 1/20 for A and to 1/10 for B-F, and the corrected expression using different prediction methods was compared to ground truth (predicting to 10 times the number of counts for B-F). A. Prediction of all UMIs in the dataset, collected into a single pool. The data was corrected from 1/20 of the reads. Ground truth is represented by black dots. B. Correction errors for different prediction methods on CPM-normalized data. The figure shows a loess fit of abs(LFC) over all genes. C-F. Scatter plots showing the correction error for each gene as the* Log_2 *fold change between corrected expression and ground truth (CPM normalized). The x axis corresponds to* Log_2 *of the number of UMIs for the gene in the downsampled data. The code to reproduce this figure is here:* [*code*]

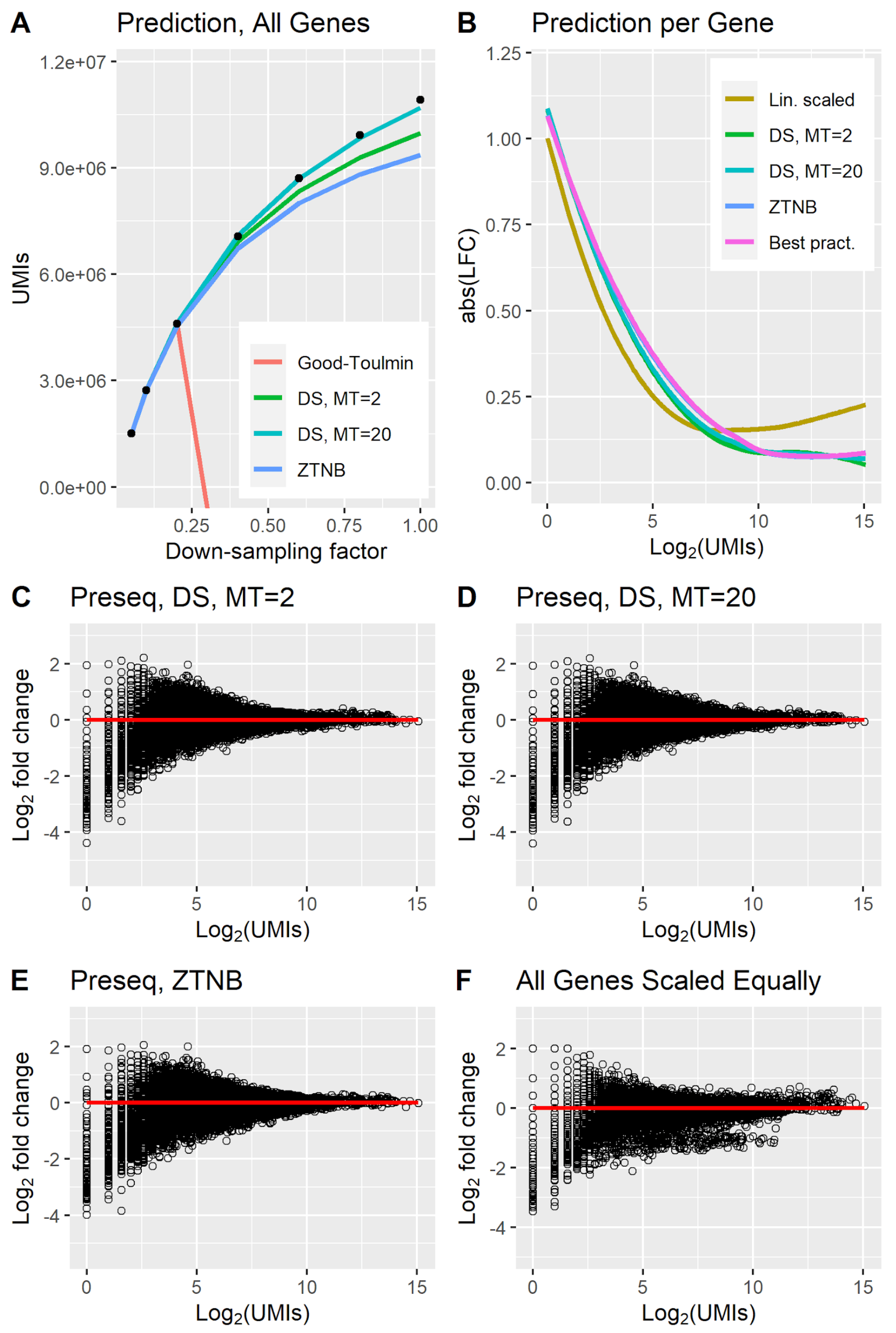

Fig S21: Correction evaluation for the MARSSEQ dataset. *The data was downsampled to 1/20 for A and to 1/10 for B-F, and the corrected expression using different prediction methods was compared to ground truth (predicting to 10 times the number of counts for B-F). A. Prediction of all UMIs in the dataset, collected into a single pool. The data was corrected from 1/20 of the reads. Ground truth is represented by black dots. B. Correction errors for different prediction methods on CPM-normalized data. The figure shows a loess fit of abs(LFC) over all genes. C-F. Scatter plots showing the correction error for each gene as the Log_2 fold change between corrected expression and ground truth (CPM normalized). The x axis corresponds to Log_2 of the number of UMIs for the gene in the downsampled data. The code to reproduce this figure is here: [code]*

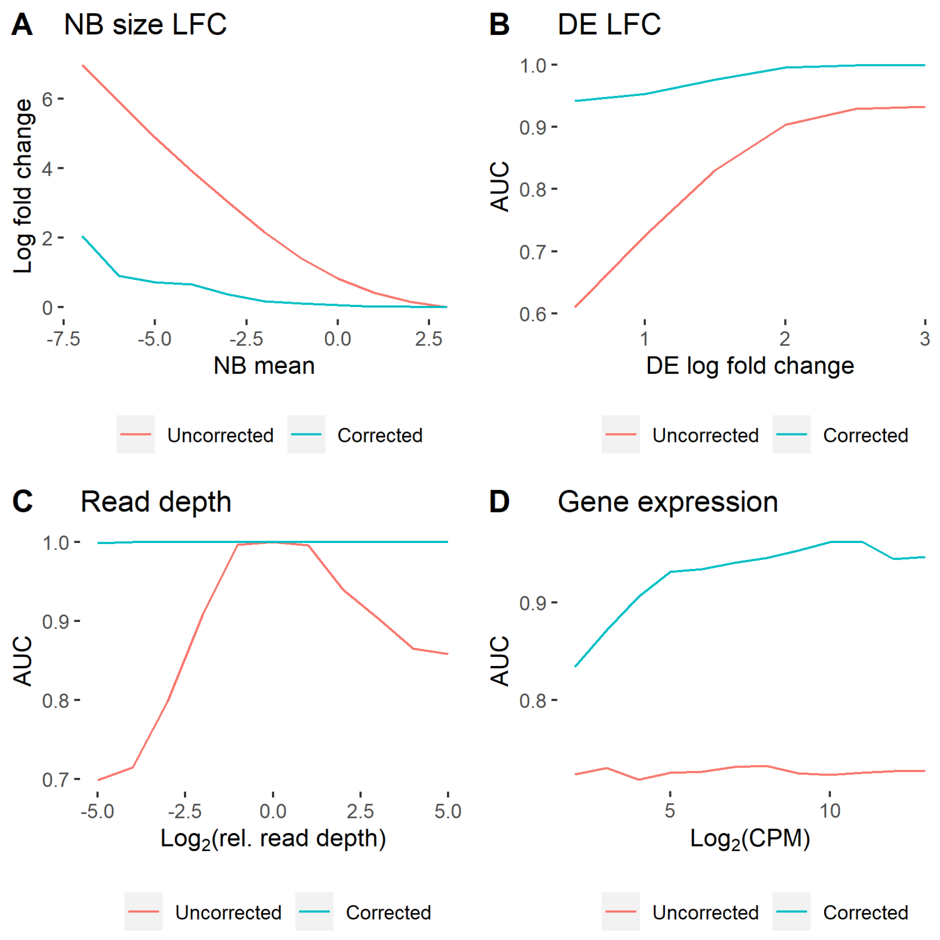

Fig S22: Theoretical investigation of the pooled amplification bias based on simulated data. *A. The log$_2$ fold change between different gene amplification values (the negative binomial mean parameter) and genes with a negative binomial mean of 3. The range of the mean variable matches that calculated from the FSCM of the PBMC_V3_3 dataset. ZTNB is used for correction. B. Investigation of the ability to from the gene expression determine if two genes are differentially expressed after the bias is added, at different levels of log$_2$ fold change between the genes. The area under the curve values are based on simulation of 5000 pairs of genes with amplification values randomly selected from that of the PBMC_V3_3 dataset. ZTNB is used for correction. C. Investigation of the ability to from the gene expression determine if a gene is differentially expressed (simulated with a log$_2$ fold change of 1) across two batches with different read depth. The negative binomial mean parameter of the amplification in the second batch is multiplied by a factor, as represented by the x axis. The area under the curve values are based on simulation of 5,000 genes with amplification values randomly selected from that of the PBMC_V3_3 dataset. Binomial downsampling (Methods) is used for correction. D. Investigation of the ability to from the gene expression determine if two genes are differentially expressed (simulated with a log$_2$ fold change of 1) after the bias is added, at different gene expression levels. The area under the curve values are based on simulation of 5000 pairs of genes with amplification values randomly selected from that of the PBMC_V3_3 dataset in a dataset with 5,000 cells at 12,000 molecules per cell (leading to on average roughly 7,400 detected molecules per cell). ZTNB is used for correction.The code to reproduce this figure is here: code*

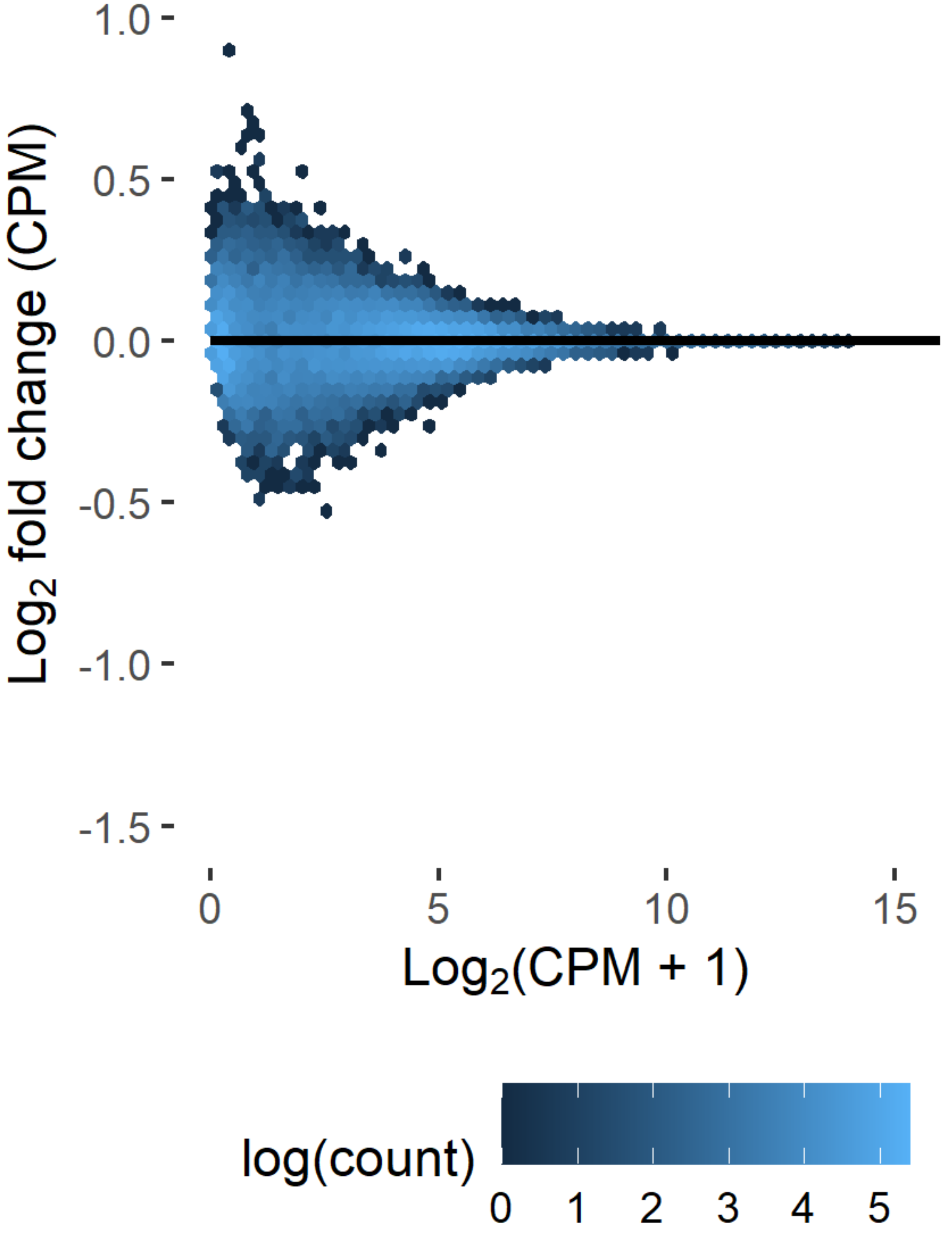

Fig S23: Sampling noise from downsampling. *Downsampled data compared to data downsampled using binomial downsampling, for which there is no sampling noise. No amplification bias is present here, since the gene expressions compared have the same number of reads per cell. This provides a bound on the accuracy possible with correction of unseen molecules in downsampled data.The code to reproduce this figure is here: code*

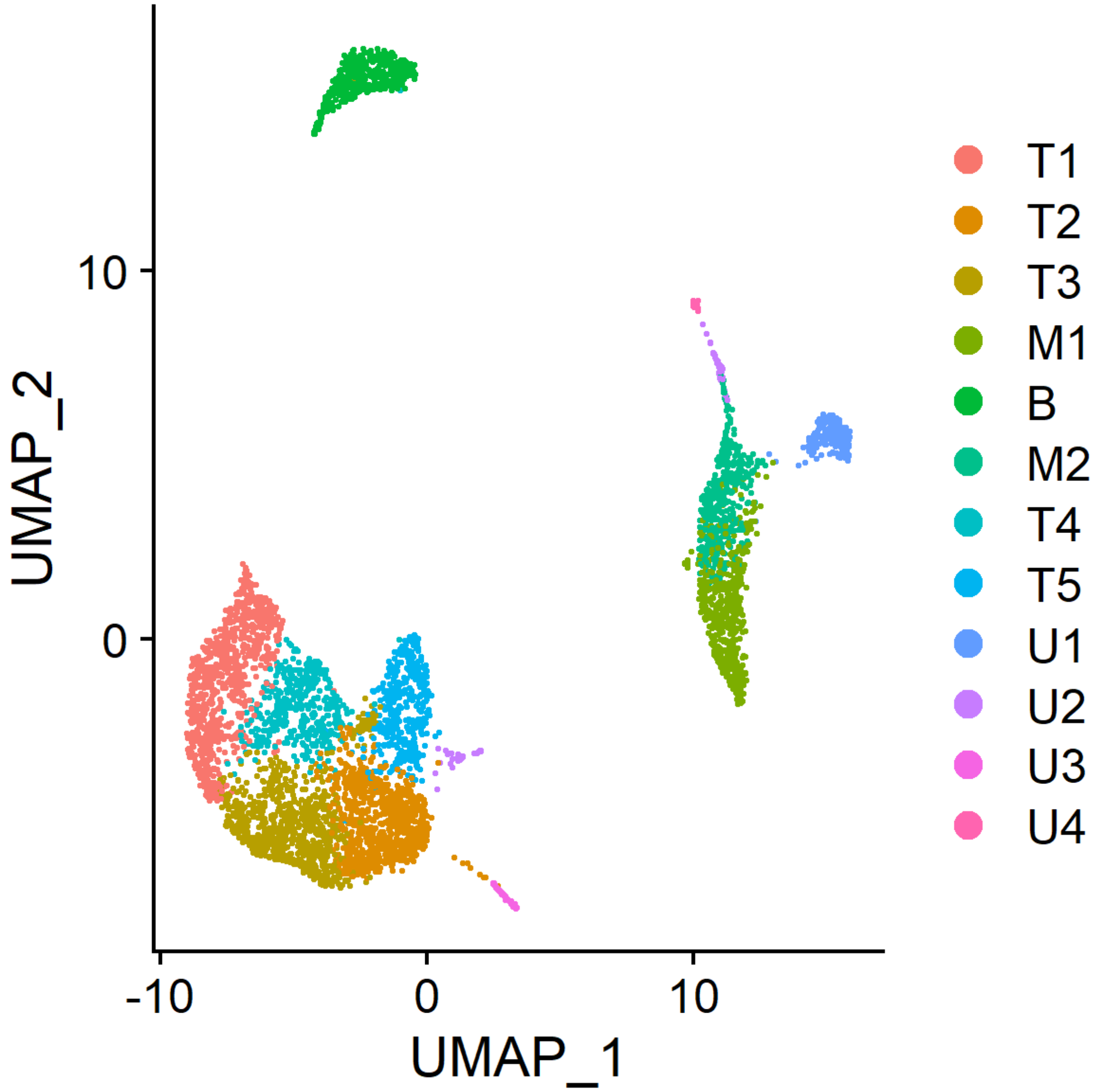

Fig S24: Clustering of EVALPBMC. *Visualization of the clusters used in Figure 5 C -- H in the main text. Cluster T1-T5 are T cells / NK cells (as determined by CD3E expression), cluster B is B cells (as determined by CD19 expression), and cluster M1-M2 are monocytes (as determined by LYZ expression). Cluster U1-U4 are unused since they contain too few cells. No code notebook is available for this figure.*

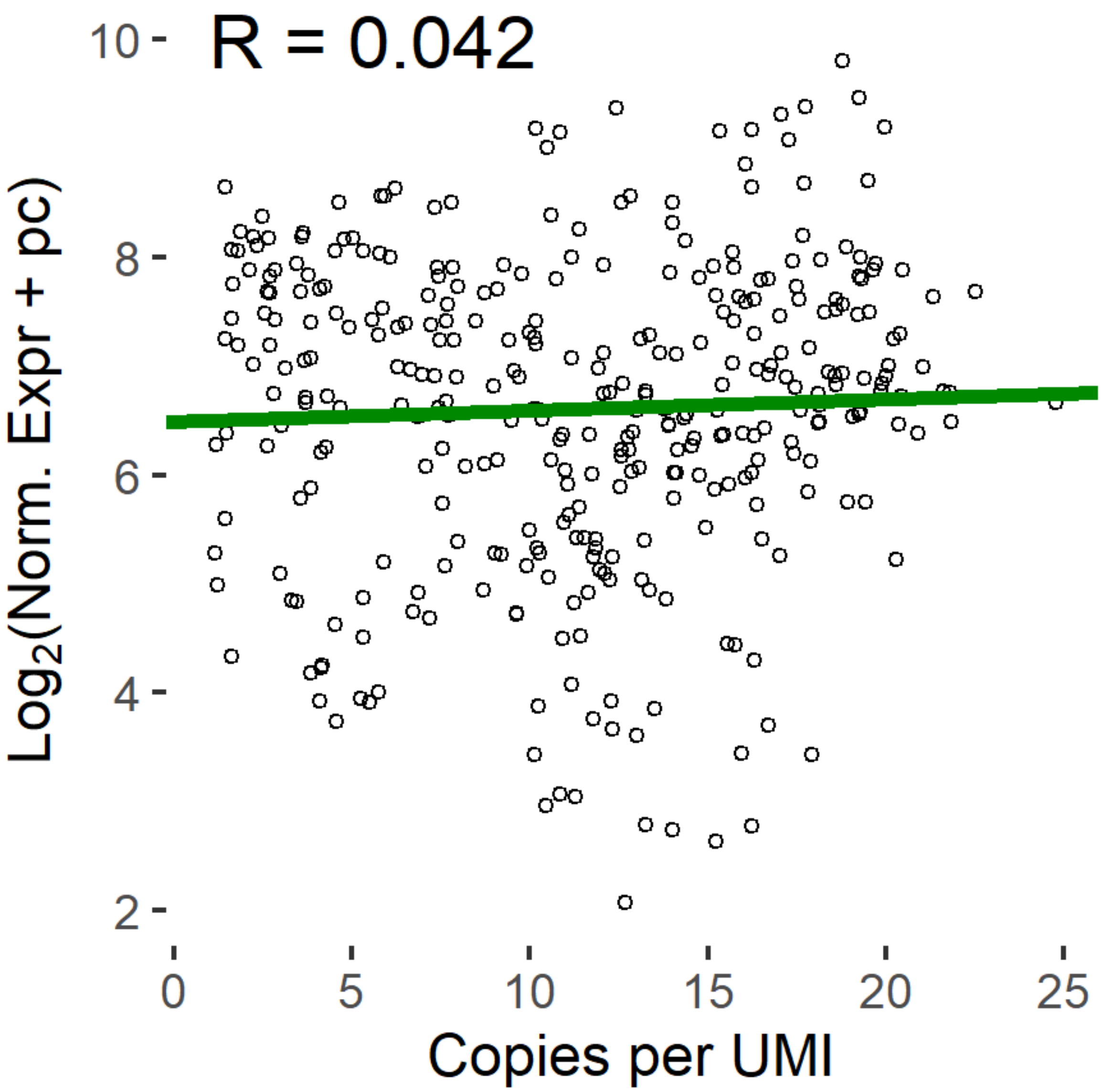

Fig S25: Amplification across clusters, corrected using ZTNB. *Similar to Figure 5 H in the main text, with the difference that the correction is done using ZTNB. No code notebook is available for this figure.*

Supplementary Tables

Table S1: FSCM values for human and mouse genes. *This table is provided as a separate file: Additional file 2 - Table S1.xlsx. The file contains FSCM values for human and mouse genes from 10X Chromium and Drop-Seq. We note that the 10X Chromium values are weighted means from several datasets (where the weight is the number of UMIs the FSCM value was calculated from in the dataset) with different saturation levels. The code to reproduce this table is here: code*

Id	Description	Technology	Reference
EVAL	Mouse brain	10x Chromium, v2	Ding et al (1), GSE132044, Cortex 1
EVALPBMC	Human PBMC	10x Chromium, v2	Ding et al (1), GSE132044, PBMC 1
EVALPBMC_DS	Human PBMC, same sample as EVALPBMC	Drop-Seq	Ding et al (1), GSE132044, PBMC 1
EVALPBMC_SW	Human PBMC, same sample as EVALPBMC	Seq-Well	Ding et al (1), GSE132044, PBMC 1
PBMC_V3	Human PBMC	10x Chromium, v3	Available at 10x Genomics' home page (2). Protein data not used.
PBMC_V3_2	Human PBMC	10x Chromium, v3	Available at 10x Genomics' home page (3). Protein data not used.
PBMC_V3_3	Human PBMC	10x Chromium, v3	Available at 10x Genomics' home page (4).
PBMC_NG	Human PBMC	10x Chromium, NetxGEM	Available at 10x Genomics' home page (5). Protein data not used.
PBMC_NG_2	Human PBMC, same sample as PBMC_V3_3.	10x Chromium, NextGEM	Available at 10x Genomics' home page (6).
PBMC_V2	Human PBMC	10x Chromium, v2	Available at 10x Genomics' home page (7).
LC	Lung tumor	10x Chromium, v2	Lambrechts et al (8). The data is available in ArrayExpress under the ascension E-MTAB-6149. Data for Patient 3, combined edge, middle and core of the tumor, was used.
MRET	Mouse retina	Drop-Seq	Macosko et al (9), GSE63473, P14 mouse retina 7.
MRET2	Mouse retina	10x Chromium, v2	Clark et al (10), GSE117614, sample p0.
MARSSEQ	Mouse ES cells and embryonic fibroblasts	MARS-seq 2.0	Keren-Shaul et al (11), samples AB339-AB343, AB435, AB415, AB416

Table S2: List of datasets used in this study.

Id	Cells (k)	Tot Cnts (M)	Tot UMIs (M)	Avg CU	Avg UMIs per Cell (k)	Avg Cnts per Cell (k)	Tot FSCM
EVAL	1.6	30	3.6	8.30	2.3	19.2	0.277
EVAL_PBMC	5.2	112	8.7	12.9	1.7	21.6	0.147
EVAL_PBMC_DS	6.9	87	4.0	21.8	0.59	12.7	0.199
EVAL_PBMC_SW	7.2	32	5.3	6.05	0.73	4.4	0.391
PBMC_V3	5.4	64	30	2.11	5.6	11.8	0.453
PBMC_V3_2	8.2	144	35	4.12	4.3	17.5	0.199
PBMC_V3_3	5.2	173	42	4.16	7.9	33.0	0.225
PBMC_NG	5.6	69	31	2.19	5.6	12.2	0.435
PBMC_NG_2	5.4	155	41	3.81	7.6	28.9	0.248
PBMC_V2	4.9	180	16	11.0	3.4	36.9	0.131
LC	21.1	305	70	4.36	3.3	14.4	0.267
MRET	20.2	68	15	4.67	0.72	3.4	0.367
MRET2	12.5	51	24	2.09	1.93	4.1	0.440
MARSSEQ	4.9	34	11	3.10	2.25	7.0	0.421

Table S3: Statistics for the datasets used in the study. *FSCM is the fraction of single-copy molecules.*

Supplementary Notes

Supplementary Note 1 - Evaluation of Prediction Algorithms

We evaluated 2 different algorithms for prediction - the Preseq DS method based on rational function approximation (RFA), and the zero truncated negative binomial (ZTNB), where the a negative binomial distribution is fitted to the CU histogram and then used for prediction. The Preseq DS method is evaluated with 2 different parameterizations (MT = 2 and MT = 20), corresponding to a different number of copies per UMI at which the CU histogram is truncated. In addition, we evaluated a selection method recommended by Deng et al (12), referred to as “best practice”, in which Preseq DS (MT = 2) is chosen for genes where the CV of the counts per UMI is greater than one and ZTNB otherwise.

We downsampled 14 datasets to one tenth of the original amount of reads, and predicted up to the original gene expression. Fig. S7 A shows the $\log_2$ fold change (LFC) between corrected expression and ground truth for genes as a function of the number of UMIs available for the gene in the downsampled dataset. The LFC presented for each method was calculated as a LOESS fit of all genes from all datasets included in this study. Lin. scaled corresponds to the case where all genes are scaled equally (i.e. no advanced prediction is used). Fig. S7 B shows an analogous analysis, with the difference that the data has been CPM-normalized before the LFC is calculated. It is evident that CPM normalization improves the correction performance.

ZTNB and best practice are almost identical, suggesting that the ZTNB is chosen for most genes for the best practice case algorithm, and we see no benefit using the best practice method. In general, ZTNB has better performance for highly expressed genes, while Preseq DS is marginally better for the middle range genes, but the overall performance is similar. Evaluation for each of the 14 datasets individually is available in Fig. S8 -- S21. In general, the results are similar across datasets. The individual evaluation of datasets show that Preseq DS is more stable when CU histograms are truncated at 2 as compared to 20; the latter parametrization sometimes gives rise to outlier genes with larger prediction errors (e.g. Fig S19 D). Interestingly, the Preseq DS algorithm (MT = 20) performs best for predicting the total number of molecules in most cases (e.g. Fig. S7 A). We speculate that this method can take advantage of the high number of molecules, and that a negative binomial distribution may not be sufficient to describe the CU histogram of differently amplified pooled genes.

Supplementary Note 2 - Evaluation of Synthetic Data

To investigate the theoretical amplification bias caused by incomplete sequencing of cDNA libraries, we generated simulated data where the number of copies of each molecule was drawn from a negative binomial distribution, with the parameters mean (μ) and size. The size parameter was set to 1 for all simulations, which is a reasonable number compared to observed real values for well amplified genes (where the fit for this parameter is reliable). The mean variable is varied throughout the different simulations. To simulate realistic values for the mean variable in some simulations, we estimated μ from the observed FSCM of the PBMC_V3_3 dataset. We first calculated a vector *f* of theoretical values for FSCM from a vector of μ values (*m*), as

$$FSCM \ = \frac{pdf(1,\mu, size)}{1 - pdf(0,\mu, size)}$$

where $pdf(x,\mu,\ size)$ is the probability density function for *x* counts for the negative binomial distribution. The value of μ for each gene in the dataset was then estimated by calculating FSCM for the gene and mapping that value to a μ value using linear interpolation between the vectors *f* and *m*.

Count matrices and CU histograms per gene were generated from simulated molecules, where molecules with zero copies were discarded. The simulation was based on 12,000 molecules per cell, which resulted in approximately 7,400 detected molecules per cell on average when using the negative binomial mean per gene as calculated from the PBMC_V3_3 dataset. For all simulations pairs of genes with different properties were generated, where each pair was compared in different ways. In each simulation 5,000 cells and 5,000 gene pairs were used. We then estimated results for two cases: uncorrected and corrected data.

In Fig. S22 A, 11 simulations were run, where μ of the first gene in each pair was set to 2^3, and μ of the second gene was varied between 2^{-7} and 2^3. The average $\log_2$ fold change for the pairs was then calculated for each simulation. All genes were expressed at 1000 CPM. Correction was done using ZTNB.

The data for Fig. S22 B was generated using 6 simulations, where the μ values of both genes in each pair were randomly selected from the PBMC_V3_3 dataset. 50% of the gene pairs were differentially expressed with a fixed $\log_2$ fold change (LFC) within each simulation, varying from 0.5 to 3. To investigate to what extent the bias affects the ability to identify gene pairs that were differentially expressed, we calculated the LFC of all gene pairs after the bias (and correction) was applied. We then based on the LFC and ground truth (i.e. if gene pairs are differentially expressed or not) calculated the area under the curve (AUC) for the Receiver Operating Characteristic (ROC) curve, which describes the ability to correctly classify gene pairs as differentially expressed. All genes were expressed at 1000 CPM. Correction was done using ZTNB.

For Fig. S22 C, we generated data using 11 simulations. The μ of the first gene in each pair was randomly selected from the PBMC_V3_3 dataset, while the μ of the second was set to the that of the first, multiplied with a factor *a*, which is constant through each simulation. The

first and second genes in the pairs are envisioned to be the same gene, but from two different datasets (1 and 2) with similar amplification pattern across genes, but different saturation levels (i.e. number of reads per molecule). *a* represents the relative saturation of dataset 2 compared to dataset 1, where dataset 2 has a times as many reads per dataset as dataset 1. The factor *a* is varied from 2^{-5} and 2^{5} through the 11 simulations. In a similar way as for Fig. S22 B, 50% of the gene pairs are differentially expressed (LFC = 1) and the AUC is calculated as a metric of the ability to correctly classify differentially expressed gene pairs. All genes were expressed at 1000 CPM. Correction was done using binomial downsampling.

In Fig. S22 D, we generated data using 12 simulations with varying gene expression, which affects the noisiness of the CU histograms used for prediction. The μ values of both genes in each pair were randomly selected from the PBMC_V3_3 dataset. The gene expression was varied from 2^{2} to 2^{13} through the simulations. In a similar way as for Fig. S22 B, 50% of the gene pairs are differentially expressed (LFC = 1) and the AUC is calculated as a metric of the ability to correctly classify differentially expressed gene pairs. Correction was done using ZTNB.

References

1. Ding J, Adiconis X, Simmons SK, Kowalczyk MS, Hession CC, Marjanovic ND, et al. Systematic comparison of single-cell and single-nucleus RNA-sequencing methods. Nature Biotechnology. 2020 Apr 6;1–10.
2. 5k_pbmc_protein_v3 -Datasets -Single Cell Gene Expression -Official 10x Genomics Support [Internet]. [cited 2020 Apr 24]. Available from: https://support.10xgenomics.com/single-cell-gene-expression/datasets/3.0.2/5k_pbmc_protein_v3
3. pbmc_10k_protein_v3 -Datasets -Single Cell Gene Expression -Official 10x Genomics Support [Internet]. [cited 2020 Apr 24]. Available from: https://support.10xgenomics.com/single-cell-gene-expression/datasets/3.0.0/pbmc_10k_protein_v3
4. 5k_pbmc_v3 -Datasets -Single Cell Gene Expression -Official 10x Genomics Support [Internet]. [cited 2020 Apr 24]. Available from: https://support.10xgenomics.com/single-cell-gene-expression/datasets/3.0.2/5k_pbmc_v3
5. 5k_pbmc_protein_v3_nextgem -Datasets -Single Cell Gene Expression -Official 10x Genomics Support [Internet]. [cited 2020 Apr 24]. Available from: https://support.10xgenomics.com/single-cell-gene-expression/datasets/3.1.0/5k_pbmc_protein_v3_nextgem
6. 5k_pbmc_v3_nextgem -Datasets -Single Cell Gene Expression -Official 10x Genomics Support [Internet]. [cited 2020 Apr 24]. Available from: https://support.10xgenomics.com/single-cell-gene-expression/datasets/3.0.2/5k_pbmc_v3_nextgem
7. pbmc4k -Datasets -Single Cell Gene Expression -Official 10x Genomics Support [Internet]. [cited 2020 Apr 24]. Available from: https://support.10xgenomics.com/single-cell-gene-expression/datasets/2.1.0/pbmc4k
8. Lambrechts D, Wauters E, Boeckx B, Aibar S, Nittner D, Burton O, et al. Phenotype molding of stromal cells in the lung tumor microenvironment. Nature Medicine. 2018 Aug;24(8):1277–89.
9. Macosko EZ, Basu A, Satija R, Nemesh J, Shekhar K, Goldman M, et al. Highly Parallel Genome-wide Expression Profiling of Individual Cells Using Nanoliter Droplets. Cell. 2015 May 21;161(5):1202–14.
10. Clark BS, Stein-O'Brien GL, Shiau F, Cannon GH, Davis-Marcisak E, Sherman T, et al. Single-Cell RNA-Seq Analysis of Retinal Development Identifies NFI Factors as Regulating Mitotic Exit and Late-Born Cell Specification. Neuron. 2019 Jun 19;102(6):1111-1126.e5.
11. Keren-Shaul H, Kenigsberg E, Jaitin DA, David E, Paul F, Tanay A, et al. MARS-seq2.0: an experimental and analytical pipeline for indexed sorting combined with single-cell RNA sequencing. Nature Protocols. 2019 Jun 1;14(6):1841–62.
12. Deng C, Daley T, Calabrese P, Ren J, Smith AD. Estimating the number of species to attain sufficient representation in a random sample. arXiv:160702804 [stat] [Internet]. 2018 May 15 [cited 2020 Apr 21]; Available from: http://arxiv.org/abs/1607.02804
