# Supplementary material for: BUTTERFLY: addressing the pooled amplification paradox with unique molecular identifiers in single-cell RNA-seq
Source: Genome Biol. 2021 Jun 8;22:174. doi: 10.1186/s13059-021-02386-z (PMC8188791; doi:10.1186/s13059-021-02386-z)
Supplement: Supplementary file 3 — Additional file 3. Review history. [file 13059_2021_2386_MOESM3_ESM.docx]

Review History

**First round of review**

**Reviewer 1**

**Were you able to assess all statistics in the manuscript, including the appropriateness of statistical tests used?**

Yes: All the scripts are deposited in Github and it was possible to assess them.

**Were you able to directly test the methods?**

Yes

**Comments to author:**

In this manuscript the authors have proposed a method to correct the relative gene abundances for amplification bias accounting for the missing molecules. This method is implemented within kallisto and bustools called BUTTERFLY. While the idea and implementation is useful, I have some concerns about the limited applicability of the method.

Major comments:

The authors claimed that the amplification bias is a property of the gene (which is independent of pooled or individual amplification). However, they highlight this as an issue of "pooled amplification". It is not very clear from the claims how is this an issue of "pooled amplification".

The variability in FSCM depends largely on the complexity and sequencing depth. Therefore, directly comparing the variance in FSCM scores across the methods is of little value here. For example, if a method 1 manages to capture 1000 mRNA molecules per cell, and then you amplify and obtain 20,000 reads after sequencing. You might actually observe most UMIs even in presence of high amplification bias, and your FSCM score would be low. Whereas, a method 2 that captures 100,000 molecules per cell, then sequencing the same amount of reads would lead to a very high FSCM.

I would like to see the effect of such correction on a linear amplification methods such as MARS-seq as a sanity check for the method.

Apart from the application of the method on actual datasets, I would like to see the impact of such correction on the power to detect relative abundances. I suggest showing the difference in the power by simulating scRNA-seq reads under different parameter combinations of captured molecule complexity, amplification bias, read coverage and differential expression effect sizes.

I would like to see by how far is the prediction off for the lowly expressed genes. On what basis the threshold of 200 molecules per gene determined?

The discussion should be expanded to include how many and which genes are highly affected by the amplification bias and if these genes share any specific properties like GC content, length etc.

Minor comments:

The graphical illustration (figure 1A) of the method is a little confusing. While I understand that the amplification is shown as a random event here, it would be easier to read if the molecules after amplification were also organised in a linear fashion as shown before the amplification. In this way the variability is clearly visible. Moreover, marking the molecules of different cells corresponding to the table would be good.

I would suggest splitting the figure 1A and B given the order of their reference in the text and especially the A is a graphical illustration of the method problem and B is the implementation with an example from a real dataset. It is confusing to refer to a dataset that is not yet mentioned in the text.

The term FSCM (Fraction of Single Copy Molecules) is not clearly defined in the text.

The figure 4A only has one "black" line while the legends suggest there should be red (10x) and blue (dropseq) dataset concordance.

**Reviewer 2**

**Were you able to assess all statistics in the manuscript, including the appropriateness of statistical tests used?**

Yes: No further review of statistical tests is needed.

**Were you able to directly test the methods?**

No

**Comments to author:**

Gustafsson et al. present a new method, called BUTTERFLY, to address incomplete sampling of mRNA molecules in sequencing libraries in the context of droplet-based single-cell RNA-seq (scRNA-seq). Although their observations and the proposed correction may be of interest for the single-cell community, there are two major problems in the current version of the manuscript that lessen my enthusiasm for this paper, namely, (1) the author fail to demonstrate that the "pooled amplification paradox" is a widespread issue in real single-cell studies with important consequences on the interpretation of the results; and (2) the presentation of the method is brief and the results of the current paper are not enough to demonstrate that the method effectively corrects the problem (or that it is better than alternative methods). I will give more details on both issues below.

Major point 1. While the authors do a good job at describing what is the pooled amplification paradox, they failed to demonstrate that failure to address it has serious consequences in real scRNA-seq datasets.

1.1 The authors' main result is that by down-sampling real scRNA-seq data, the relative abundance of two genes may appear switched, as illustrated very well in Figure 1B. However, this result is based on downsampling real datasets, in some cases by a lot (e.g., retaining only 10% of reads as in Figure 3). Is it reasonable to expect that real experiments will be carried out at 1/10th of the sequencing depth of the studies chosen by the authors?

1.2 In principle, the analysis carried out in Figure 4 is more important, provided that the authors are able to show increased concordance across technology after butterfly correction. Figure 4 in its present form, however, fails to convincingly show this. In fact, it is risky to base the conclusion on extrapolation of a non-linear relationship, as this is very often an inaccurate process. Furthermore, there are clear issues with Figure 4A, e.g. the legend mentions two curves, while only one is shown.

Major point 2. If I understand correctly, the authors have re-implemented several methods developed in ecology. While there is a brief description of the relevant literature, there is almost no detail about the methods themselves. I do not think it is fair to assume that the readership of Genome Biology will be familiar with the relevant literature, nor that they will go look up the details in the cited papers. I think it is paramount for the authors to describe in greater details the implemented methods (with assumptions and formulas) in the Methods section.

2.1 Is BUTTERFLY just another name for ZTNB in the context of scRNA-seq? Or did the author modify the approach in some way? Or is BUTTERFLY a method that includes ZTNB estimation as one of its steps? More details in the methods section are required.

2.2 I found Figure 3 underwhelming: it is true that the correction leads to more symmetric fold-changes between downsampled and original data, but does it lead to smaller absolute log-fold-changes? Furthermore, the increase in CCC does not appear dramatic from 0.981 to 0.997 and one could argue that the CCC value of the uncorrected data is already quite high.

2.3 In the Discussion, the authors state that they have shown "that BUTTERFLY can help reduce batch effect between datasets sequenced at different depths". I do not believe that they have shown that. Actually showing this would be a major point in favour of their method and I suggest that the authors look more into demonstrating this, perhaps by showing a joint analysis of two or more datasets from different technologies and/or depths.

Minor issues:

- Page 9, line 45: the authors state that "the improvement in concordance is driven by individual genes", what would be the alternative? What do they mean exactly by individual genes here?

- Page 12, "Correction for unseen molecules" section: what does the acronym "CU" stand for? Similarly what is "DS"? And "MT" in the next paragraph?

- Page 13, "quantile" normalisation may refer to all-quantile matching or one-quantile matching (third quartile?). More details and a reference are needed.

- Page 14., line 17: Pearson correlation does not measure linearity, but linear correlation. - Page 14., line 17: a reference is needed for CCC.

**Authors Response**

**Point-by-point responses to the reviewers’ comments:**

Reviewer #1: In this manuscript the authors have proposed a method to correct the relative gene abundances for amplification bias accounting for the missing molecules. This method is implemented within kallisto and bustools called BUTTERFLY. While the idea and implementation is useful, I have some concerns about the limited applicability of the method.

*We thank the reviewer for taking the time to evaluate the manuscript and for providing valuable comments. Below we have responded to each of the specific concerns. To address the concern about the limited applicability of the method, we have added an analysis showing the effectiveness of batch correction using amplification data (Figure 5 A-B). In addition, we added an analysis showing the presence of amplification bias across cell types within the same gene, and how BUTTERFLY correction can help to address that bias (Figure 5 C-H).*

Major comments:

The authors claimed that the amplification bias is a property of the gene (which is independent of pooled or individual amplification). However, they highlight this as an issue of "pooled amplification". It is not very clear from the claims how is this an issue of "pooled amplification".

*The amplification bias is indeed a property of the gene or a group of molecules within a gene (for example within a cell type, as we show in figure 5). The problem with having different amplification across genes comes into play when cDNA molecules corresponding to distinct genes are pooled together prior to amplification and subsequent sequencing. In that case, most of the reads will be associated with the highly copied genes, while the lowly copied genes will be diminished in abundance. If all lowly copied genes were amplified and sequenced in a separate batch (which is challenging technically), these genes would have as many reads as the others, and the problem we are addressing would not exist (or at least to a much lesser extent). This is the reason why we call it the pooled amplification paradox. We have added a comment about this in the introduction.*

*The variability in FSCM depends largely on the complexity and sequencing depth. Therefore, directly comparing the variance in FSCM scores across the methods is of little value here. For example, if a method 1 manages to capture 1000 mRNA molecules per cell, and then you amplify and obtain 20,000 reads after sequencing. You might actually observe most UMIs even in presence of high amplification bias, and your FSCM score would be low. Whereas, a method 2 that captures 100,000 molecules per cell, then sequencing the same amount of reads would lead to a very high FSCM.*

*It is true that the FSCM metric for genes within a dataset depends on the dataset saturation (i.e number of reads per molecule). Changing the saturation for a dataset will, however, change the FSCM metric for all genes in the same direction. Still, it is true that the variation in FSCM across genes seems to increase with saturation. Compare for example EVALPBMC (highly saturated) with PBMC_V3 (lowly saturated). This effect can be explained by the fact that it is practically impossible to saturate the genes with high FSCM - their FSCM value does not change much when more reads are added, unless a very large amount of reads are added. The FSCM for the other genes, however, is more affected, stretching out the histogram. This effect can thus lead to a problematic comparison of the variation in amplification across genes when compared across datasets with different degrees of saturation. But the question is what effect this has on our conclusion, that the variation in amplification across genes is larger in 10X Chromium data. We compare the copies per UMI values from two pairs of datasets with one 10X and one Drop-Seq dataset: (EVALPBMC, EVALPBMC_DS) and (MRET2, MRET). As can be seen in table S2, the Drop-Seq datasets are the most saturated within each pair (the CU column, representing copies per UMI). Yet, if we look at the histograms in Supplementary Figure S1/S2, the variation in FSCM is higher for the 10X datasets. So, although we recognize that there can be a problem in comparing datasets of different saturation levels and that the results should be interpreted with care, we still believe it is of value to report this result. We have added some text in the results emphasizing that the results should be interpreted with care.*

*Another related concern is that genes with high FSCM values may be filtered in datasets with few total UMIs. We previously filtered the genes with fewer than 200 UMIs to avoid uncertainty in the FSCM estimation. This limit was changed to 30 to include more genes with high FSCM. As seen in the figures and the presented numbers, the effect is still the same, with more variation in FSCM for 10X Chromium datasets.*

I would like to see the effect of such correction on a linear amplification methods such as MARS-seq as a sanity check for the method.

*We have added a MARS-seq 2.0 dataset analysis to Supplementary Figure 3, 4, and 5, and added Supplementary Figure 21. As expected, the variation in the MARS-seq data is fairly low compared to other datasets. We note that MARS-seq still uses PCR in the second library amplification step, although you are correct that the preamplification step uses linear amplification.*

Apart from the application of the method on actual datasets, I would like to see the impact of such correction on the power to detect relative abundances. I suggest showing the difference in the power by simulating scRNA-seq reads under different parameter combinations of captured molecule complexity, amplification bias, read coverage and differential expression effect sizes.

*We have added Supplementary Figure 22, where we have simulated data based on the assumption that the number of copies of each molecule follows a negative binomial distribution. We have chosen to not use a standard differential expression analysis tool for this purpose, but instead look at how pairs of genes, where some have different “real” expressions, can be identified using the average expression levels. The reason for this is that although we correct data, there will still be a difference between the genes, the correction is not perfect. If this difference is detected or not using statistical methods, such as DESeq2 or Seurat, will depend on a lot of factors, such as the number of cells etc., which are not of interest for this analysis. The balance between false positives and false negatives is better explained by the AUC (of the ROC curve).*

I would like to see by how far is the prediction off for the lowly expressed genes. On what basis the threshold of 200 molecules per gene determined?

*We assume this concerns old Figure 4 (now Figure 4 D-E). The 200 molecules was a typo, the correct number is 100 per gene. As shown in Supplementary figure 7B, the prediction worsens the fewer molecules the gene has since the histograms become noisy - how far the predictions are off for the different methods and number of UMIs can directly be seen in that figure. When we predict two datasets to make them more similar, prediction errors from both datasets will affect the correlation. As can be seen in Supplementary figure 7, the prediction error is roughly half (a little bit more) of that of the uncorrected data at around 100 molecules. We reasoned that this was a suitable limit that also turned out to work practically. A text about this has been added to the Figure Details section in Methods.*

*The discussion should be expanded to include how many and which genes are highly affected by the amplification bias and if these genes share any specific properties like GC content, length etc.*

*We added Supplementary Figure 6 which shows the dependence between FSCM / Copies per UMI and GC content / Average transcript length. We also added Supplementary Table 1, in which the FSCM values for human and mouse genes are available.*

Minor comments:

The graphical illustration (figure 1A) of the method is a little confusing. While I understand that the amplification is shown as a random event here, it would be easier to read if the molecules after amplification were also organised in a linear fashion as shown before the amplification. In this way the variability is clearly visible. Moreover, marking the molecules of different cells corresponding to the table would be good.

*The figure is now changed as requested. The non-mixed molecules are now linearly ordered and the outline of the molecules now have a color that corresponds to the cell.*

I would suggest splitting the figure 1A and B given the order of their reference in the text and especially the A is a graphical illustration of the method problem and B is the implementation with an example from a real dataset. It is confusing to refer to a dataset that is not yet mentioned in the text.

*We have split the figure into two figures.*

The term FSCM (Fraction of Single Copy Molecules) is not clearly defined in the text.

*We added a small section about FSCM in the methods.*

The figure 4A only has one "black" line while the legends suggest there should be red (10x) and blue (dropseq) dataset concordance.

*This is a mistake, the figure has been corrected. The concordance is simply one line.*

Reviewer 2

Reviewer #2: Gustafsson et al. present a new method, called BUTTERFLY, to address incomplete sampling of mRNA molecules in sequencing libraries in the context of droplet-based single-cell RNA-seq (scRNA-seq). Although their observations and the proposed correction may be of interest for the single-cell community, there are two major problems in the current version of the manuscript that lessen my enthusiasm for this paper, namely, (1) the author fail to demonstrate that the "pooled amplification paradox" is a widespread issue in real single-cell studies with important consequences on the interpretation of the results; and (2) the presentation of the method is brief and the results of the current paper are not enough to demonstrate that the method effectively corrects the problem (or that it is better than alternative methods). I will give more details on both issues below.

*We thank the reviewer for taking the time to evaluate the manuscript and for providing valuable comments. Below we have responded to each of the specific concerns. To address the first concern mentioned above, we have added an analysis showing the effectiveness of batch correction using amplification data (Figure 5 A-B). In addition, we show the presence of amplification bias across cell types within the same gene, and how BUTTERFLY correction can help to address that bias (Figure 5 C-H). To address the second question, we in addition to the two figures mentioned also added an analysis of simulated data, for which our correction methods clearly helps in identifying gene pairs with different expression levels (Supplementary Figure 22).*

Major point 1. While the authors do a good job at describing what is the pooled amplification paradox, they failed to demonstrate that failure to address it has serious consequences in real scRNA-seq datasets.

*We have added Figure 5 A-B, which shows the effectiveness of batch correction using amplification data, and Figure 5 C-H, which shows the presence of the amplification bias across cell types within the same gene, and how BUTTERFLY correction can help to address that bias.*

1.1 The authors' main result is that by down-sampling real scRNA-seq data, the relative abundance of two genes may appear switched, as illustrated very well in Figure 1B. However, this result is based on downsampling real datasets, in some cases by a lot (e.g., retaining only 10% of reads as in Figure 3). Is it reasonable to expect that real experiments will be carried out at 1/10th of the sequencing depth of the studies chosen by the authors?

*We evaluate in total 14 datasets by downsampling to a tenth of the reads. It is true that for some of these datasets, downsampling to 10% of the reads yields an unusually lowly saturated dataset. This is however not true for all of these datasets. For example, the datasets EVALPBMC and EVALPBMC_DS are heavily saturated (although many molecules are still not detected), and downsampling these datasets to 10% of the reads yields datasets with depth in the range of published data. EVALPBMC_DS has on average 21.8 copies per molecule, so the dataset at 1/10 of the reads will have at least 2.18, but in practice substantially more since many lowly copied molecules are lost in the downsampling. This can be compared to the PBMC_V3 dataset that has 2.11 copies per molecule. The aim with this figure is to show that the gene expression is improved when predicting up to 10 times the original amount of reads, and that predicting to at least to such an extent leads to an improvement of the data. We also added an analysis of synthetic data (Supplementary Figure 22), where we investigate the effectiveness of correction at different saturation levels. We also note that there have been recent analyses arguing for shallow sequencing as an optimal tradeoff between more reads / cell vs. more cells, and as datasets increase in size with respect to numbers of cells, we expect further decreases in depth of sequencing (see e.g., Zhang, Ntranos and Tse, “Determining sequencing depth in single-cell RNA-seq experiment”, Nature Communications, 2020).*

1.2 In principle, the analysis carried out in Figure 4 is more important, provided that the authors are able to show increased concordance across technology after butterfly correction. Figure 4 in its present form, however, fails to convincingly show this. In fact, it is risky to base the conclusion on extrapolation of a non-linear relationship, as this is very often an inaccurate process. Furthermore, there are clear issues with Figure 4A, e.g. the legend mentions two curves, while only one is shown.

*We added a batch correction figure (Figure 5 A-B). Here, we applied binomial downsampling (Methods), which is a more effective method than prediction when the goal is to batch correct two batches. This figure, together with CCC values, shows that correcting for this bias helps in making datasets more similar. Furthermore, we added analyses of simulated data, which show that correction clearly improves the ability to determine if two genes are differentially expressed. The old figure 4 was incorrect and has been corrected.*

Major point 2. If I understand correctly, the authors have re-implemented several methods developed in ecology. While there is a brief description of the relevant literature, there is almost no detail about the methods themselves. I do not think it is fair to assume that the readership of Genome Biology will be familiar with the relevant literature, nor that they will go look up the details in the cited papers. I think it is paramount for the authors to describe in greater details the implemented methods (with assumptions and formulas) in the Methods section.

*We have reimplemented the Good-Toulmin estimator in R and the ZTNB method in bustools in C++. For the results in the paper, we however use a slightly modified version of the ZTNB implementation available in PreseqR. For the preseq DS method, we use the implementation in PreseqR. To further clarify the methods, we have added text to the methods describing these methods in more detail.*

2.1 Is BUTTERFLY just another name for ZTNB in the context of scRNA-seq? Or did the author modify the approach in some way? Or is BUTTERFLY a method that includes ZTNB estimation as one of its steps? More details in the methods section are required.

*We added a section in Methods called “Overview of the BUTTERFLY method” that clarifies the scope of BUTTERFLY. As is now described in methods, BUTTERFLY is the name of the correction method that uses prediction of unseen species together with library size normalization of the pooled gene expression across cells to provide gene abundance estimates. BUTTERFLY is thereby not just limited to ZTNB, but also includes two other existing estimators, Preseq DS and Good-Toulmin. As part of the extended content of this submission, BUTTERFLY now also includes binomial downsampling for batch correction. However, in the bustools software, we have only implemented ZTNB, much due to the similar performance of ZTNB and PreSeq DS.*

2.2 I found Figure 3 underwhelming: it is true that the correction leads to more symmetric fold-changes between downsampled and original data, but does it lead to smaller absolute log-fold-changes? Furthermore, the increase in CCC does not appear dramatic from 0.981 to 0.997 and one could argue that the CCC value of the uncorrected data is already quite high.

*The absolute log-fold-changes can be seen in Supplementary Figure 7A. The absolute error is however of less interest; the BUTTERFLY correction includes a CPM normalization after the prediction, which effectively removes such errors. It is true that the increase in CCC may appear insignificant. There are however three things to consider: 1) Although the correction may be small, it can still have a large impact on for example the UMAP projection of datasets, as shown in Figure 5 A-B. 2) The bias is much larger for some genes than for others. While most genes are not heavily affected, some are. For those genes, the correction is important. 3) The reason why we only predict 10 times in this figure is that downsampling to a higher degree discards a large portion of the data and creates unrealistic datasets, leaving little to work with for the predictions. The whole bias, which would be corrected for if we would predict to infinity, is expected to be much larger. This can be seen in our experiments using simulated data (Supplementary Figure 22). Figure 4D also gives a hint about the changes in expression over a larger saturation range for 3 genes in two datasets.*

2.3 In the Discussion, the authors state that they have shown "that BUTTERFLY can help reduce batch effect between datasets sequenced at different depths". I do not believe that they have shown that. Actually showing this would be a major point in favour of their method and I suggest that the authors look more into demonstrating this, perhaps by showing a joint analysis of two or more datasets from different technologies and/or depths.

*We have added a new figure (Figure 5 A-B), which shows batch correction of two similar datasets with different sequencing depths. For this purpose, we also developed a new flavor of the method, called binomial downsampling (see Methods). For batch correction, where the amplification can be assumed to be similar across genes, it is better to “predict” what the gene expression of the more saturated dataset would look like given less reads instead of applying prediction to the less saturated dataset. The main advantage of this approach is that this type of prediction is a much less difficult problem to solve. Although our correction may seem small (from CCC=0.991 to 0.994), it still has quite an effect on the UMAP projection, as can be seen in the figure.*

Minor issues:

- Page 9, line 45: the authors state that "the improvement in concordance is driven by individual genes", what would be the alternative? What do they mean exactly by individual genes here?

*This text has been changed to instead state that some genes are more affected than others by this bias (and correction).*

- Page 12, "Correction for unseen molecules" section: what does the acronym "CU" stand for? Similarly what is "DS"? And "MT" in the next paragraph?

*We have added explanations of these terms.*

- Page 13, "quantile" normalisation may refer to all-quantile matching or one-quantile matching (third quartile?). More details and a reference are needed.

*We have edited the methods to clarify what we are doing and have added a reference for clarification.*

- Page 14., line 17: Pearson correlation does not measure linearity, but linear correlation. - Page 14., line 17: a reference is needed for CCC.

*We changed the wording, and added a reference.*

**Second round of review**

**Reviewer 1**

The authors have answered all my questions. I have no more concerns.

**Reviewer 2**

The revised version of the manuscript is improved in many aspects and the authors addressed all my comments. In particular, the methods section now more clearly describe the BUTTERFLY method, the new Figure 5 offers a compelling case for the utility of the correction, and the new simulation study offers a way to better understand the results.

There are still a few minor points for the authors to address.

- Although it is explained in the methods, it would be nice to have a one-sentence intuition in the results section about what FSCM measures and why it is useful to look at FSCM across datasets as a way to quantify the amplification bias.

- I’m still unconvinced by the use of the concordance correlation coefficient (CCC) as a way to evaluate the effectiveness of the correction. In fact, the increase in CCC between uncorrected and corrected data is modest, mainly because the CCC is already very high for uncorrected data. However, visually, the plots in Figure 4A, B, C show a much higher degree of correction than what is expected from comparing the CCC values. Can the authors use a different/additional metric to quantify the correction? For instance, the mean squared error of the log-fold-change estimator?

- Is there a reason why the version of bustools used for this paper is not part of the official release? Are there plans to merge the butterfly branch of bustools into the main branch?

- The authors use different algorithms for different analyses, e.g., they use the DS algorithm in Figure 4 C, 5 F, and 5 H, and the ZTNB algorithm for most other analyses, which begs the question: when is it better to use one or the other? A paragraph discussing the pros and cons of each approach should be added to the Discussion.

- The inline greek letters in the Methods section appear as white squares in the text.

**Authors Response**

**Point-by-point responses to the reviewers’ comments:**

Although it is explained in the methods, it would be nice to have a one-sentence intuition in

the results section about what FSCM measures and why it is useful to look at FSCM across datasets as a way to quantify the amplification bias.

*We added a sentence introducing FSCM and its rationale in the very beginning of the results section.*

I’m still unconvinced by the use of the concordance correlation coefficient (CCC) as a way to evaluate the effectiveness of the correction. In fact, the increase in CCC between uncorrected and corrected data is modest, mainly because the CCC is already very high for uncorrected data. However, visually, the plots in Figure 4A, B, C show a much higher degree of correction than what is expected from comparing the CCC values. Can the authors use a different/additional metric to quantify the correction? For instance, the mean squared error of the log-fold-change estimator?

*We have added MSE in addition to CCC as suggested for figure 4 A-C and 5 A-B, and extended the CCC description in methods to also include MSE. As pointed out, the MSE numbers show a larger difference between corrected and uncorrected data. We do think theCCC is useful as well as it measures directly not only whether there is a linear relationship but an identical relationship, so we have left it in place alongside the MSE.*

Is there a reason why the version of bustools used for this paper is not part of the official release? Are there plans to merge the butterfly branch of bustools into the main branch?

*The functions will be distributed in the main branch of bustools in the next release which is imminent and will precede publication of the paper. Specifically, the predict function in the prediction branch of bustools will be integrated into the master branch.*

The authors use different algorithms for different analyses, e.g., they use the DS algorithm in Figure 4 C, 5 F, and 5 H, and the ZTNB algorithm for most other analyses, which begs the question: when is it better to use one or the other? A paragraph discussing the pros and cons of each approach should be added to the Discussion.

*We have added a paragraph discussing the two methods in the discussion.*

The inline greek letters in the Methods section appear as white squares in the text.

*Thanks for noting this. It is likely an artifact introduced by the conversion from MS Word to PDF in the submission system; it does not appear that way in the Word file. Since we think the production will use the Word file, we suspect that this issue will be resolved automatically but we will keep an eye on it.*
